# Supplementary material for: Small molecule induced STING degradation facilitated by the HECT ligase HERC4
Source: Nat Commun. 2024 May 29;15:4584. doi: 10.1038/s41467-024-48922-w (PMC11137104; doi:10.1038/s41467-024-48922-w)
Supplement: Supplementary file 1 — Supplementary Information [file 41467_2024_48922_MOESM1_ESM.pdf]

Supplementary Information

Small molecule induced STING degradation facilitated by the HECT ligase HERC4

Merve Mutlu[1,#], Isabel Schmidt[1], Andrew I. Morrison[1,3], Benedikt Goretzki[1], Felix Freuler[1], Damien Begue[1], Oliver Simic[1], Nicolas Pythoud[1], Erik Ahrne[1], Sandra Kapps[1], Susan Roest[1], Debora Bonenfant[1,4], Delphine Jeanpierre[1], Thi-Thanh-Thao Tran[1], Rob Maher[2], Shaojian An[2], Amandine Rietsch[1], Florian Nigsch[1], Andreas Hofmann[1], John Reece-Hoyes[2,5], Christian N. Parker[1], Danilo Guerini[1]

[1]Novartis BioMedical Research, Basel, Switzerland

[2]Novartis BioMedical Research, Cambridge, MA, USA

[3]Current address: Amsterdam UMC location Vrije Universiteit Amsterdam, Molecular Cell Biology & Immunology, Amsterdam institute for Infection and Immunity, De Boelelaan 1117, Amsterdam, The Netherlands

[4]Current address: Monte Rosa Therapeutics, Basel, Switzerland

[5]Current address: Vector Biology, Cambridge, MA, USA

[#]Corresponding author: merve.koch@novartis.com

Suppplementary Figures……………………………………………………………………………….…..3
Supplementary Figure 1. Compound characterization of AK59 and QK50……………………………..…..3
Supplementary Figure 2. Schematic representation of the STING constructs that were transiently expressed in HEK293T cells………………….………………………………………….…………….…..…..5
Supplementary Figure 3. CRISRP-Cas9 genome-wide screening results….……………….…………….…..6
Supplementary Figure 4. Validation of *HERC4, UBA5* and *UBA6* as genes responsible for AK59 activity on STING expression.………………………..……………………………….…………………...……...7
Supplementary Figure 5. Loss of HERC4 reduces compound-dependent IRF pathway inhibition….…….…..8
Supplementary Figure 6. Effect of AK59 in *HERC4* or *STING* knockout backgrounds……………………….9
Supplementary Figure 7. Interaction of HERC4 and STING in the presence of AK59 treatment……..….…10
Supplementary Methods…………………………………………………………………………………....11
Synthesis of 5-iodoquinolin-8-amine……………………………………………………………………...11
Synthesis of 5-(pyridin-3-yl)quinolin-8-amine……………………………………………………………… 11
Synthesis of N-(5-(pyridin-3-yl)quinolin-8-yl)-4-(trifluoromethyl)benzenesulfonamide AK59………………11
Synthesis of N-(4-bromonaphthalen-1-yl)-4-(trifluoromethyl)benzenesulfonamide………………………… 12
Synthesis of N-(4-(pyridin-3-yl)naphthalen-1-yl)-4-(trifluoromethyl)benzenesulfonamide QK59…………...12
LC-HRMS Analyses……………………………………………………………………………………...12
HR-MS……………………………………………………………………………………………………12
NMR spectra of AK59………………………………………………………………………………………14
NMR spectra of QK50………………………………………………………………………………………19
Supplementary Tables……………………………..……………………………………………………….25
Supplementary Table 1. CRISPR sgRNA sequences…..………………………………………………….25
Blots from Supplementary Figures…………………………………………………………………….26

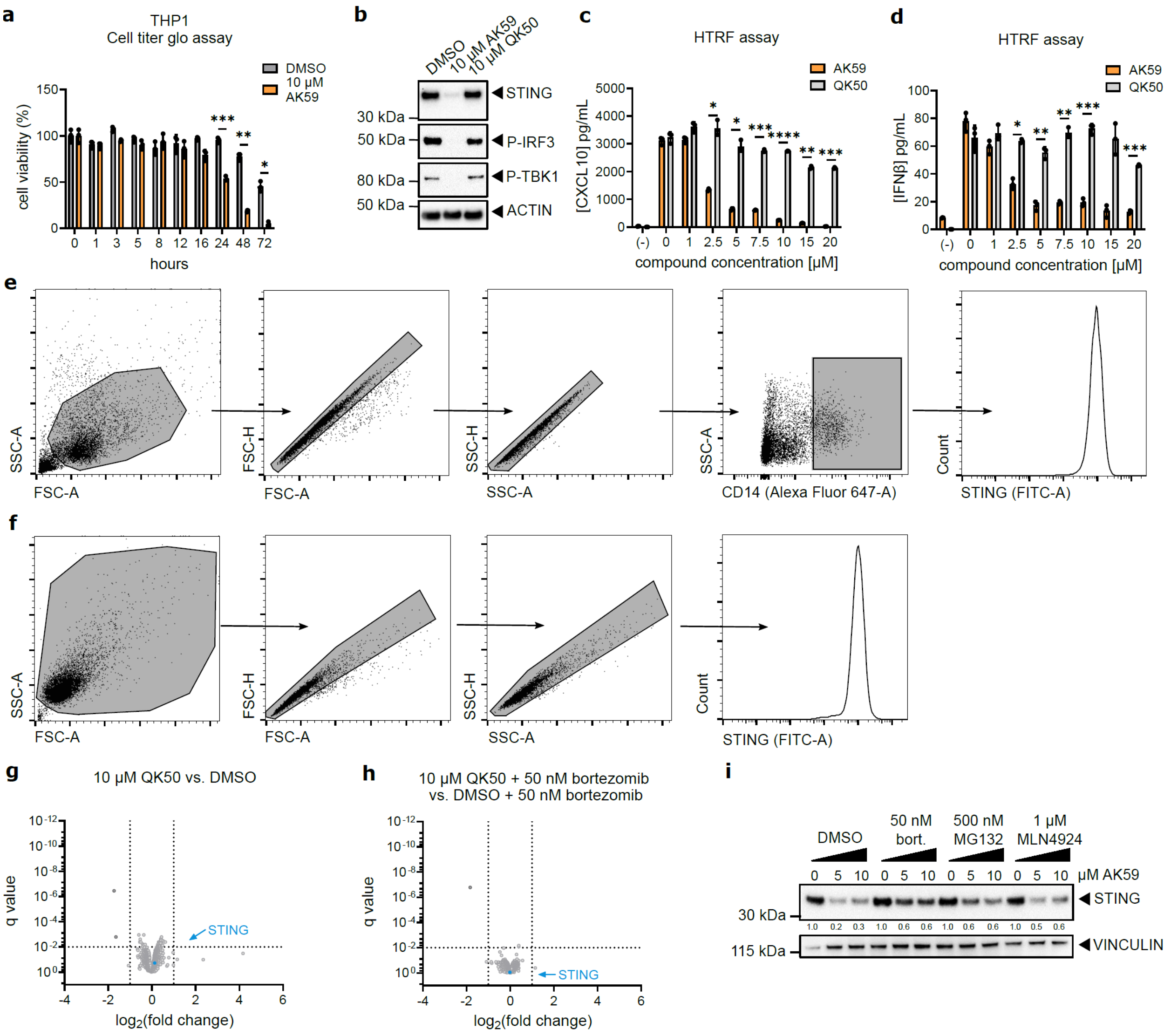

Supplementary Figure 1. Compound characterization of AK59 and QK50. a, Viability of THP1 cells upon 10 μM AK59 treatment. Luminescence reads were normalized to the control unstimulated sample. Data plotted as mean ± SD of three individual biological replicates. Significance calculated using two-way ANOVA followed by Šidák's correction. Significance indicated as * p<0.05, **p<0.01, ***p<0.001. **b,** Western blots of indicated protein expressions in cGAMP stimulated Dual-THP1 cells upon 10 μM AK59 or QK50 treatment. Results are representative of 3 independent experiments. **c,** HTRF assay measuring CXCL10 levels of cGAMP stimulated Dual-THP1 cells treated with increasing doses of either AK59 or QK50. Data plotted as mean ± SD of three individual biological replicates. Calculated half-maximal effective concentrations (EC50s) are 1.914 and 9.367 for AK59 and QK50 respectively. Significance calculated using two-way ANOVA followed by Šidák's correction. Significance indicated as * p<0.05, **p<0.01, ***p<0.001, ****p<0.0001. **d,** HTRF assay measuring IFN beta levels of cGAMP stimulated Dual-THP1 cells treated with increasing doses of either AK59 or QK50. Data plotted as mean ± SD of three individual biological replicates. Calculated half-maximal effective concentrations (EC50s) are 1.397 and 25.55 for AK59 and QK50 respectively. Significance calculated using two-way ANOVA followed by Šidák's correction. Significance indicated as * p<0.05, **p<0.01, ***p<0.001. **e,** Representative gating strategy of CD14+ (Alexa Fluor 647-A) monocytes from human PBMCs. **f,** Representative gating strategy of THP1 lines. **g-h,** Proteomic analysis of 10 μM QK50 treated THP1 cells and co-treated with **(h)** or without **(g)** 50nM of bortezomib compared to matching DMSO control samples. Significantly altered protein abundances (in red) are shown with a log2 fold change < - 1 or > 1 and a q value cutoff of 0.01. **i,** Western blot of STING protein

expression in AK59 treated THP1 cells with/without prior proteasomal inhibition (bortezomib or MG132) or neddylation inhibition (MLN4924). STING expression quantified by first normalizing to the respective loading controls and the calculation the ratio to the DMSO control. Results are representative of 3 independent experiments. Source data are provided as a Source Data file.

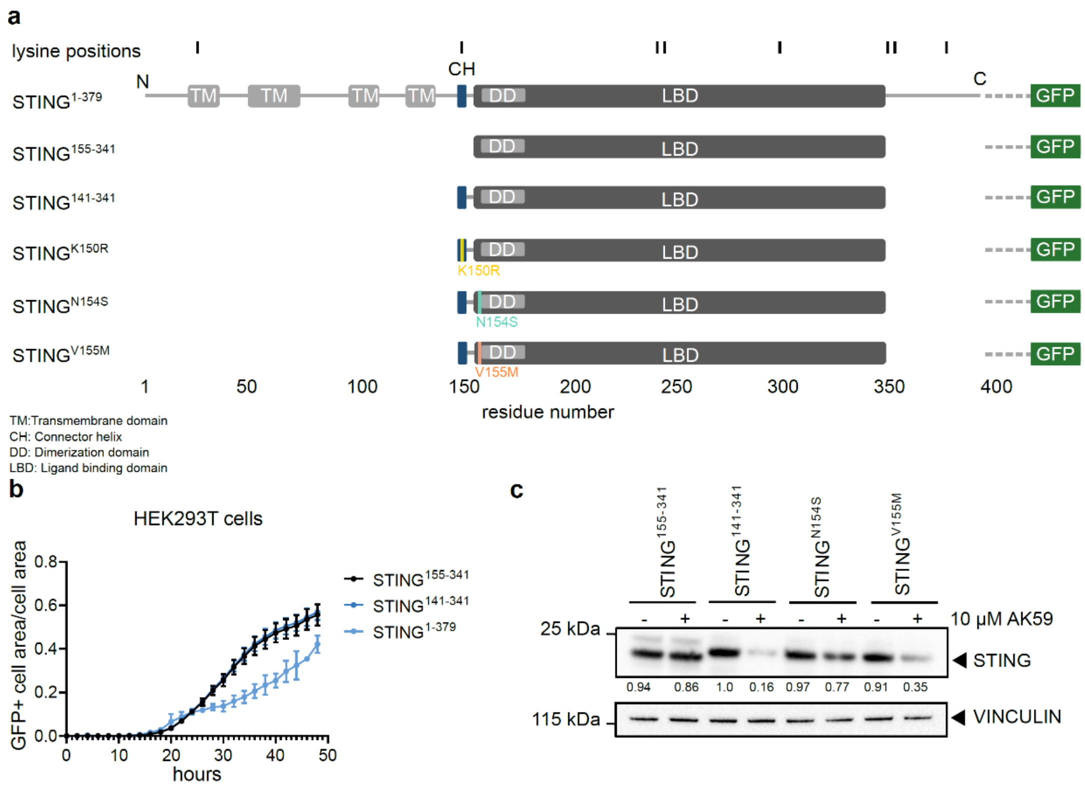

Supplementary Figure 2. Schematic representation of the STING constructs that were transiently expressed in HEK293T cells. a, Schematic representation of the STING constructs. Above the panel, lysine residues in the full-length STING represented. The position of the single point mutation K150R is indicated by yellow. The position of SAVI related single point mutations (N154S and N155M) is indicated by peach and light green respectively. **b,** Live cell tracking of C-terminally GFP-tagged STING$^{155-341}$-GFP, STING$^{141-341}$-GFP or STING$^{1-379}$-GFP constructs expressed in HEK293T cells. The data collection started from the moment of transfection up to 48h. GFP+ cell area is normalized to total cell area detected per image. For each data point 16 pictures/well were averaged per biological replicate. Three biological replicates were plotted as mean ± SD. **c,** Western blot showing STING protein levels upon AK59 treatment of HEK293T cells transiently expressing STING$^{141-341}$ and SAVI mutants STING$^{141-341_N154S}$, STING$^{141-341_V155M}$ (not GFP tagged). VINCULIN was used as a loading control. Results are representative of 3 independent experiments. Source data are provided as a Source Data file.

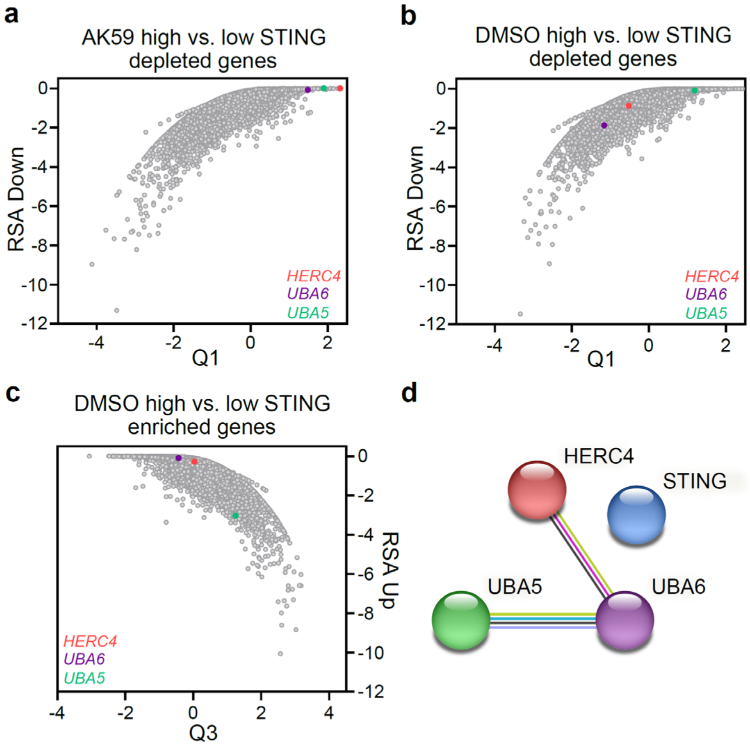

Supplementary Figure 3. CRISRP-Cas9 genome-wide screening results. a, RSA down versus Q1 values from CRISPR-Cas9 screen of 10 μM AK59 treated THP1-Cas9 cells represented in a dot plot. The comparison represented in the plot is high STING expression to low STING expression in the AK59 treatment group. Each dot represents a gene from the CRISPR-Cas9 library. **b,** RSA up versus Q3 values from CRISPR-Cas9 screen of DMSO treated THP1-Cas9 cells represented in a dot plot. The comparison represented in the plot is high STING expression to low STING expression in the DMSO treated group. Each dot represents a gene from the CRISPR-Cas9 library. **c,** RSA down versus Q1 values from CRISPR-Cas9 screen of DMSO treated THP1-Cas9 cells represented in a dot plot. The comparison represented in the plot is high STING expression to low STING expression in the DMSO treated group. Each dot represents a gene from the CRISPR-Cas9 library. **d,** STRING analysis representing the interaction between HERC4, UBA5, UBA6 and STING (STRING version 11.5). Source data are provided as a Source Data file.

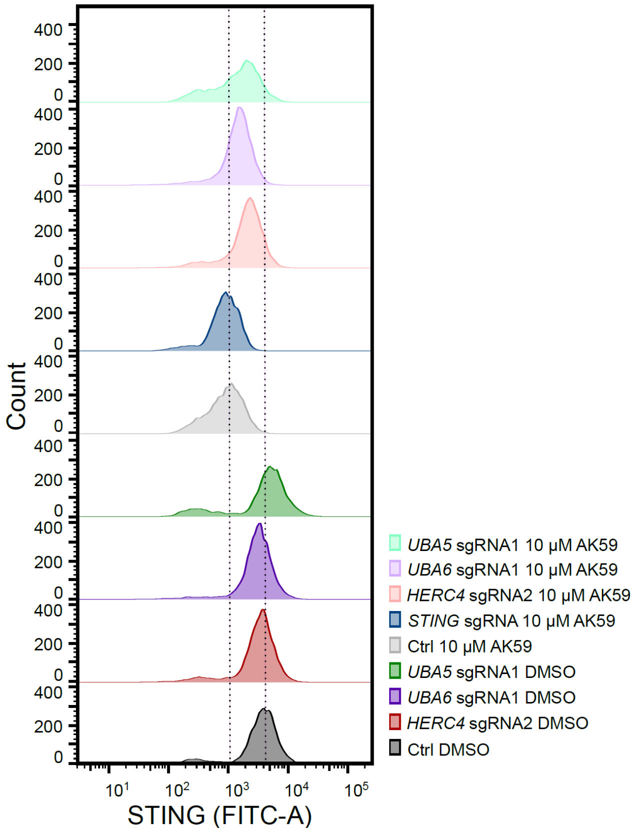

Supplementary Figure 4. Validation of *HERC4, UBA5* and *UBA6* as genes responsible for AK59 activity on STING expression. FACS analysis of STING expression on Ctrl sgRNA, *HERC4* sgRNA2, *UBA6* sgRNA1 or *UBA5* sgRNA1 transduced THP1-Cas9 cells that are treated either with DMSO or 10 µM AK59. *STING* knockout line was taken along as a negative control (blue). The dashed lines indicate the median of Ctrl DMSO treated or 10 µM AK59 treated samples. The FACS experiments performed in three biological replicates and representative FACS reads plotted using FlowJo (Version 10.6.1). Results are representative of 3 independent experiments. Source data are provided as a Source Data file.

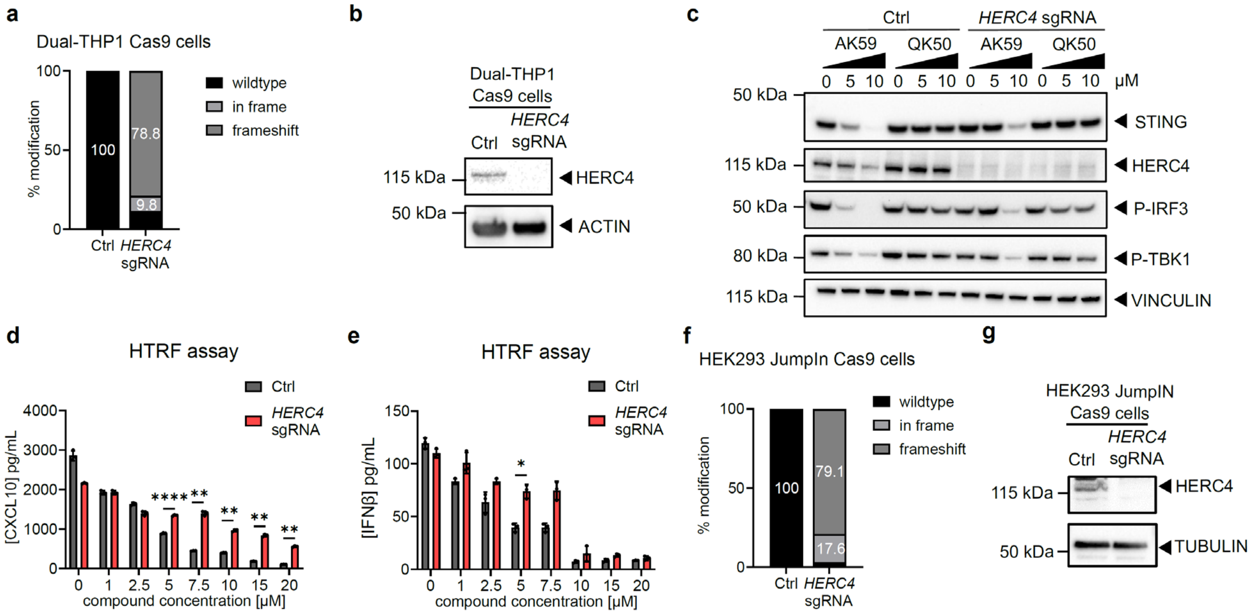

Supplementary Figure 5. Loss of HERC4 reduces compound-dependent IRF pathway inhibition. a, TIDE analysis of Ctrl sgRNA or *HERC4* sgRNA1 transduced Dual-THP1-Cas9 cells. Undefined sequencing reads were excluded. **b,** Western blot of HERC4 protein levels on Ctrl sgRNA or *HERC4* sgRNA transduced Dual-THP1-Cas9 cells. Results are representative of 3 independent experiments. **c,** Western blots of indicated protein expressions in Ctrl or *HERC4* sgRNA Dual-THP1 cells upon AK59 or QK50 treatment. Results are representative of 3 independent experiments. **d,** HTRF assay measuring CXCL10 levels of cGAMP stimulated Ctrl or *HERC4* sgRNA Dual-THP1 cells treated with increasing doses of either AK59 or QK50. Data plotted as mean ± SD of three individual biological replicates. Calculated half-maximal effective concentrations (EC50s) are 3.004 and 282.0 for Ctrl and *HERC4* sgRNA respectively. Significance calculated using two-way ANOVA followed by Šidák's correction. Significance indicated as $^{**}p<0.01$, $^{****}p<0.0001$. **e,** HTRF assay measuring IFN beta levels of cGAMP stimulated Dual-THP1 cells treated with increasing doses of either AK59 or QK50. Data plotted as mean ± SD of three individual biological replicates. Calculated half-maximal effective concentrations (EC50s) are 4.224 and 8.308 for Ctrl and *HERC4* sgRNA respectively. Significance calculated using two-way ANOVA followed by Šidák's correction. Significance indicated as $^{*}p<0.05$. **f,** TIDE analysis from Ctrl sgRNA or *HERC4* sgRNA1 transduced HEK293-JumpIN-Cas9 cells. **g,** Western blot of HERC4 protein levels on Ctrl sgRNA or *HERC4* sgRNA1 transduced HEK293-JumpIN-Cas9 cells. Results are representative of 3 independent experiments. Source data are provided as a Source Data file.

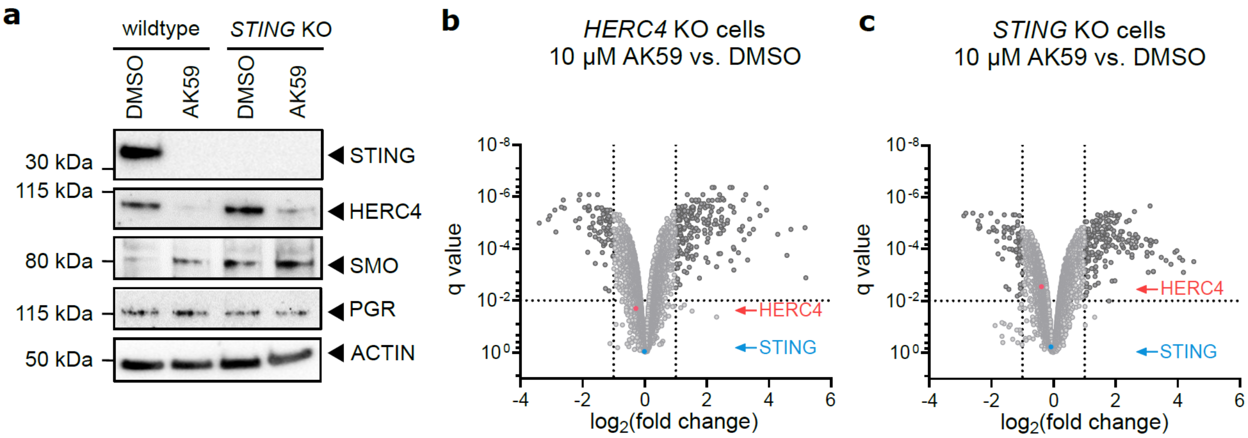

Supplementary Figure 6. Effect of AK59 in *HERC4* or *STING* knockout backgrounds. a, Western blot showing STING, HERC4, SMO and PGR protein levels in Dual-THP1 cells treated with either AK59 or DMSO (vehicle) control for 16 hours. ACTIN was used as a loading control. Results are representative of 3 independent experiments. **b-c,** Volcano plots of the proteomics analysis of either *HERC4* knockout **(b)** or *STING* knockout **(c)** Dual-THP1 cells treated with 10 μM AK59 for 16 hours compared to matching DMSO control samples. Significantly altered protein abundances (dark gray) are shown with a log2 fold change < - 1 or > 1 and a q value cutoff of 0.01. STING shown in blue and HERC4 shown in red. Source data are provided as a Source Data file.

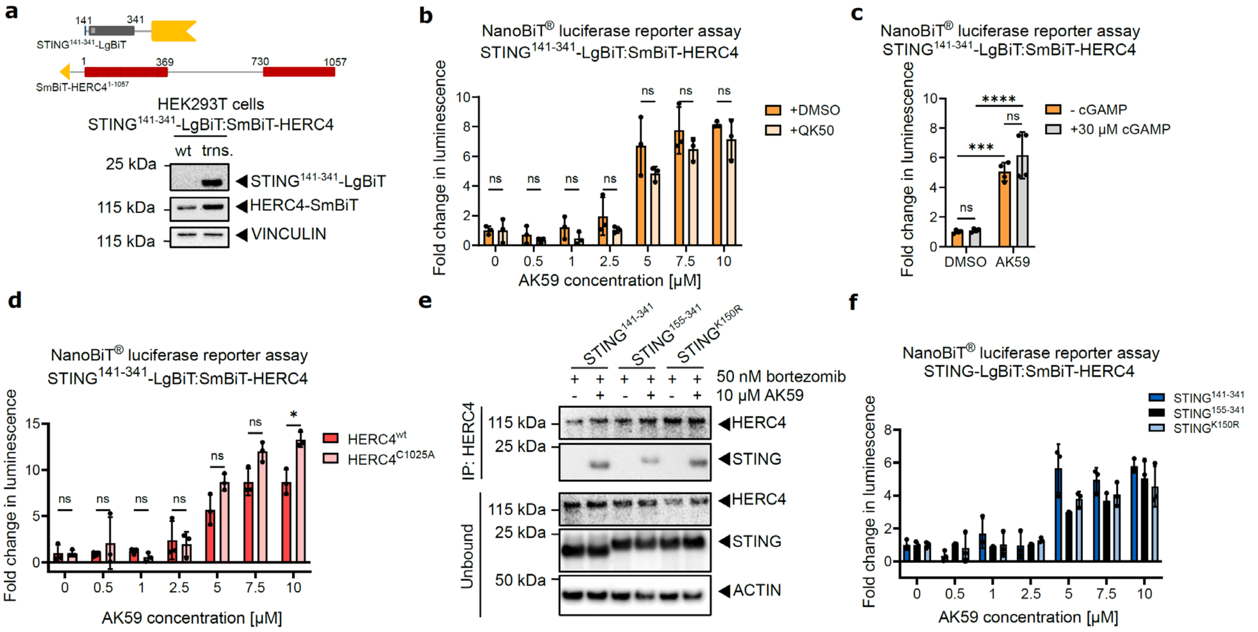

Supplementary Figure 7. Interaction of HERC4 and STING in the presence of AK59 treatment. a, Schematic representation of the NanoBiT® constructs. Western blot showing the expression of STING$^{141\text{-}341}$-LgBiT and SmBiT-HERC4$^{1\text{-}1057}$ constructs in wildtype (wt) versus transfected (trns.) HEK293T cells (below). Results are representative of 3 independent experiments. **b,** NanoBiT® assay on STING$^{141\text{-}341}$-LgBiT and SmBiT-HERC4 expressing HEK293T cells in increasing doses of AK59 and either 10 µM QK50 or DMSO control. QK50 treatment was added 3 hours prior to the AK59 treatment and kept it throughout for competition. Three biological replicates plotted as mean ± SD. Significance calculated using two-way ANOVA followed by Šidák's correction. Significance indicated as ns $p > 0.05$. **c,** NanoBiT® assay on STING$^{141\text{-}341}$-LgBiT and SmBiT-HERC4 expressing HEK293T cells in the presence of 10 µM AK59 and/or 30 µM cGAMP. Four biological replicates plotted as mean ± SD. Significance calculated using two-way ANOVA followed by Šidák's correction. Significance indicated as ns $p>0.05$, ***$p<0.001$, ****$p<0.0001$. **d,** NanoBiT® assay on STING$^{141\text{-}341}$-LgBiT and either SmBiT-HERC4^{wt} or SmBiT-HERC4^{C1025A} expressing HEK293T cells in the presence of increases doses of AK59 treatment. Three biological replicates plotted as mean ± SD. Significance calculated using two-way ANOVA followed by Šidák's correction. Significance indicated as * $p<0.05$. **e,** HERC4 pulldown followed by western blot on HEK293T cells transfected with the indicated STING expression constructs and treated with 50nM bortezomib and with either DMSO control or 10 µM AK59. Results are representative of 3 independent experiments. **f,** NanoBiT® complementation assay on various STING-LgBiT (STING$^{141\text{-}341}$-LgBiT, STING$^{155\text{-}341}$-LgBiT, STING$^{141\text{-}341\ K150R}$-LgBiT) and SmBiT HERC4 expressing HEK293T cells in the presence of 10 µM AK59. Three biological replicates plotted as mean ± SD. Statistical significance calculated using two-way ANOVA followed by Šidák's correction. Source data are provided as a Source Data file.

Supplementary Methods:

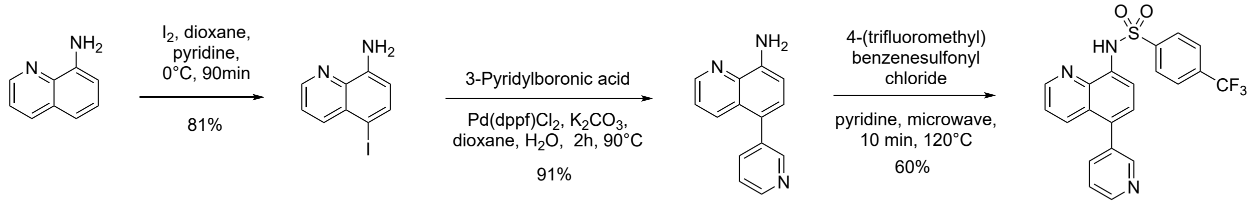

AK59

5-iodoquinolin-8-amine

A solution of quinolin-8-amine (12 g, 82 mmol) in 1.4-dioxane (500 mL) and water (500 mL) was cooled to 0°C and iodine (62.1 g, 245 mmol) was added. The reaction mixture was stirred for 90 min at 0°C, saturated aqueous sodium sulfate was added and the aq. phase was extracted with CH_2Cl_2. The combined organic phases were washed with brine, dried over anhydrous sodium sulfate, filtered and the solvent was removed in vacuo. The crude product was purified by ISCO flash-chromatography (eluent: cyclohexane/EtOAc from 0-20% EtOAc) to give the title compound (147 mg, 0.34 mmol, 84%) as a brown solid.

TLC (cyclohexane:EtOAc, 4:1 v/v): Rf = 0.37; 1H NMR (400 MHz, d6-DMSO): δ 8.72 (dd, J = 4.1, 1.6 Hz, 1H), 8.16 (s, 1H), 7.80 (d, J = 8.1 Hz, 1H), 7.60 (d, J = 4.1 Hz, 1H), 6.72 (d, J = 8.2 Hz, 1H), 6.18 (s, 2H). UPLC-MS: t_R = 1.03 min. MS (ESI): [M+H] m/z 271.0. UPLC purity: 96 %.

5-(pyridin-3-yl)quinolin-8-amine

A mixture of 5-iodoquinolin-8-amine (1.00 g, 3.70 mmol), pyridin-3-ylboronic acid (0.91 g, 7.41 mmol), $PdCl_2$(dppf) (271 mg, 0.37 mmol) and K_2CO_3 (1.54 g, 11.11 mmol) in 1,4-dioxane (6 mL) and water (1 mL) was heated for 25 h at 90°C. The reaction mixture was quenched by addition of water and the aq. phase was extracted with EtOAc. The combined organic phases were washed with brine, dried over anhydrous sodium sulfate, filtered and the solvent was removed in vacuo. The crude product was purified by ISCO flash-chromatography (eluent: cyclohexane/EtOAc from 0-100% EtOAc) to give the title compound (552 mg, 2.42 mmol, 65%) as a beige solid.

TLC (cyclohexane:EtOAc, 1:1 v/v): Rf = 0.23; 1H NMR (400 MHz, d6-DMSO): δ 8.78 (dd, J = 4.1, 1.6 Hz, 1H), 8.63 (dd, J = 2.3, 0.9 Hz, 1H), 8.59 (dd, J = 4.8, 1.6 Hz, 1H), 8.11 (dd, J = 8.6, 1.6 Hz, 1H), 7.85 (ddd, J = 7.8, 2.4, 1.7 Hz, 1H), 7.53 – 7.47 (m, 2H), 7.33 (d, J = 7.8 Hz, 1H), 6.97 (d, J = 7.9 Hz, 1H), 6.16 (s, 2H). UPLC-MS: t_R = 0.63 min. MS (ESI): [M+H] m/z 222.4. UPLC purity: 97 %.

N-(5-(pyridin-3-yl)quinolin-8-yl)-4-(trifluoromethyl)benzenesulfonamide AK59

A mixture of 5-(pyridin-3-yl)quinolin-8-amine (140 mg, 0.63 mmol) and 4-(trifluoromethyl)benzene-1-sulfonyl chloride (96 mg, 0.381 mmol) in pyridine (3 mL) was heated in a Biotage microwave reactor for 10 min at 120°C. The reaction mixture was poured into water and the aq. phase was extracted with EtOAc. The combined organic phases were washed with brine, dried over anhydrous sodium sulfate, filtered and the solvent was removed in vacuo. The crude product was purified by ISCO flash-chromatography (eluent: CH_2Cl_2 / CH_2Cl_2/MeOH 4:1 from 0-20% DCM/MeOH 4:1) to give the title compound (165 mg, 0.38 mmol, 60%) as a colorless solid.

TLC (CH_2Cl_2/MeOH 9:1 v/v): Rf = 0.61; 1H NMR (400 MHz, d6-DMSO): δ 10.60 (s, 1H), 8.89 (d, J = 4.1 Hz, 1H), 8.66 (dd, J = 7.7, 3.5 Hz, 2H), 8.17 (dd, J = 14.5, 8.3 Hz, 3H), 7.96 – 7.87 (m, 3H), 7.78 (d, J = 7.9 Hz, 1H), 7.63 – 7.52 (m, 3H). 13C NMR (400 MHz, d6-DMSO): δ 149.77, 149.49, 148.66, 143.81, 139.31, 137.67, 134.01, 133.98, 133.58, 132.78, 132.57, 131.73, 127.98, 127.98, 127.92, 126.50, 126.48, 126.22, 123.74, 122.89, 117.35. UPLC-MS: t_R = 1.15 min. MS (ESI): [M+H] m/z 430.2. UPLC purity: 99 %. HRMS (m/z): [M+H]+ calcd. for C21H14N3O2F3S, 430.08316; found, 430.08345.

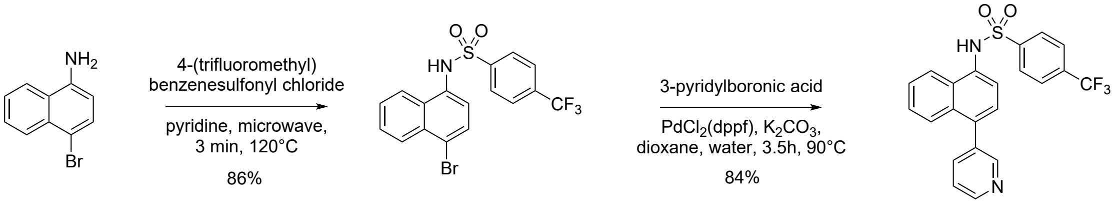

QK59

N-(4-bromonaphthalen-1-yl)-4-(trifluoromethyl)benzenesulfonamide

A mixture of 4-bromonaphthalen-1-amine (150 mg, 0.66 mmol) and 4-(trifluoromethyl)benzene-1-sulfonyl chloride (196 mg, 0.786 mmol) in pyridine (4.5 mL) was heated in a Biotage microwave reactor for 3 min at 120°C. The reaction mixture was poured into water and the aq. phase was extracted with EtOAc. The combined organic phases were washed with brine, dried over anhydrous sodium sulfate, filtered and the solvent was removed in vacuo. The crude product was purified by ISCO flash-chromatography (eluent: CH2Cl2 / CH2Cl2/MeOH 4:1 from 0-20% DCM/MeOH 4:1) to give the title compound (284 mg, 0.55 mmol, 86%) as a pink solid.

TLC (cyclohexane:EtOAc, 1:1 v/v): Rf = 0.78; 1H NMR (400 MHz, d6-DMSO): δ 10.64 (s, 1H), 8.11 (d, J = 8.4 Hz, 1H), 7.98 (d, J = 8.5 Hz, 1H), 7.93 – 7.85 (m, 4H), 7.83 (d, J = 8.0 Hz, 1H), 7.67 (ddd, J = 8.3, 6.8, 1.2 Hz, 1H), 7.53 (ddd, J = 8.3, 6.9, 1.2 Hz, 1H), 7.09 (d, J = 8.0 Hz, 1H).

UPLC-MS: tR = 1.26 min. MS (ESI): [M+H] m/z 430.0. UPLC purity: 98 %.

N-(4-(pyridin-3-yl)naphthalen-1-yl)-4-(trifluoromethyl)benzenesulfonamide QK59

A mixture of N-(4-bromonaphthalen-1-yl)-4-(trifluoromethyl)benzenesulfonamide (175 mg, 0.41 mmol), pyridin-3-ylboronic acid (100 mg, 0.81 mmol), PdCl2(dppf) (29.8 mg, 0.041 mmol) and K2CO3 (169 mg, 1.22 mmol) in 1,4-dioxane (6 mL) and water (1 mL) was heated for 3.5 h at 90°C. The reaction mixture was quenched by addition of water and the aq. phase was extracted with EtOAc. The combined organic phases were washed with brine, dried over anhydrous sodium sulfate, filtered and the solvent was removed in vacuo. The crude product was purified by ISCO flash-chromatography (eluent: cyclohexane/EtOAc from 0-100% EtOAc) to give the title compound (147 mg, 0.34 mmol, 84%) as an orange powder.

TLC (cyclohexane:EtOAc, 1:1 v/v): Rf = 0.36; 1H NMR (400 MHz, d6-DMSO): δ 10.66 (s, 1H), 8.67 (dd, J = 4.8, 1.6 Hz, 1H), 8.64 (dd, J = 2.3, 0.9 Hz, 1H), 8.10 – 8.04 (m, 1H), 7.95 (d, J = 1.1 Hz, 4H), 7.89 (dt, J = 7.8, 2.0 Hz, 1H), 7.72 – 7.67 (m, 1H), 7.58 – 7.53 (m, 1H), 7.52 – 7.47 (m, 2H), 7.42 (d, J = 7.6 Hz, 1H), 7.25 (d, J = 7.7 Hz, 1H). 13C NMR (151 MHz, DMSO-d6) δ 149.87, 148.74, 144.09, 137.34, 135.11, 135.00, 132.49 (q, J = 32.4 Hz, 1C), 132.16, 131.67, 129.76, 127.71 (s, 2C), 127.16, 127.08, 126.57 (q, J = 3.6 Hz, 2C), 126.30, 125.23, 123.52 (s, 2C), 123.15, 123.43 (q, J = 272.9 Hz, 1C). UPLC-MS: tR = 1.07 min. MS (ESI): [M+H] m/z 429.5. UPLC purity: 99 %. HRMS (m/z): [M+H]+ calcd. for C22H15N2O2F3S, 429.08791; found, 429.08813

Waters UPLC Acquity; column: Acquity HSS T3, 1.8µm, 2.1*50mm at 60°C, Eluent A: water + 0.05 % HCOOH + 3.75 mM ammonium acetate, B: ACN + 0.04 % HCOOH, Gradient: 5 to 98 % B in 1.4 min, Flow: 1.0 mL/min).

LC-HRMS Analyses

The analyses were performed by using electrospray ionization in positive ion modus after separation by liquid chromatography (Ultimate 3000 from Dionex). The elemental composition was derived from the mass spectra acquired at the high resolution of about 70’000 on an Q Exactive Plus mass spectrometer (Thermo Scientific). The high mass accuracy below 2 ppm was obtained by using a lock mass.

HR-MS

LC/ESI-MS and LC-UV data were recorded using a Thermo Scientific Q Exactive Plus mass spectrometer equipped with an electrospray ionization source and coupled to a Thermo Ultimate 3000 liquid

chromatograph equipped with a diode array detector. The instrument was lock mass calibrated with the protonated di-octylphtalate ion (m/z 391.28429). The accurate mass was obtained by averaging 6 scans at a mass resolution of ca 70000 (FWHM definition). The mass accuracy of the system has been found to be better than 2 ppm. The chromatography was performed at 150 µL/min flow rate (1 mm C18-column) with a polar gradient from 5% to 100% acetonitrile in 5 min. 0.05 % and 0.04% formic acid was used as the modifier additive in the mobile phases water and acetonitrile, respectively.

Sample was prepared in 1mg/ml in ACN:H2O (7:3).

NMR spectra of AK59

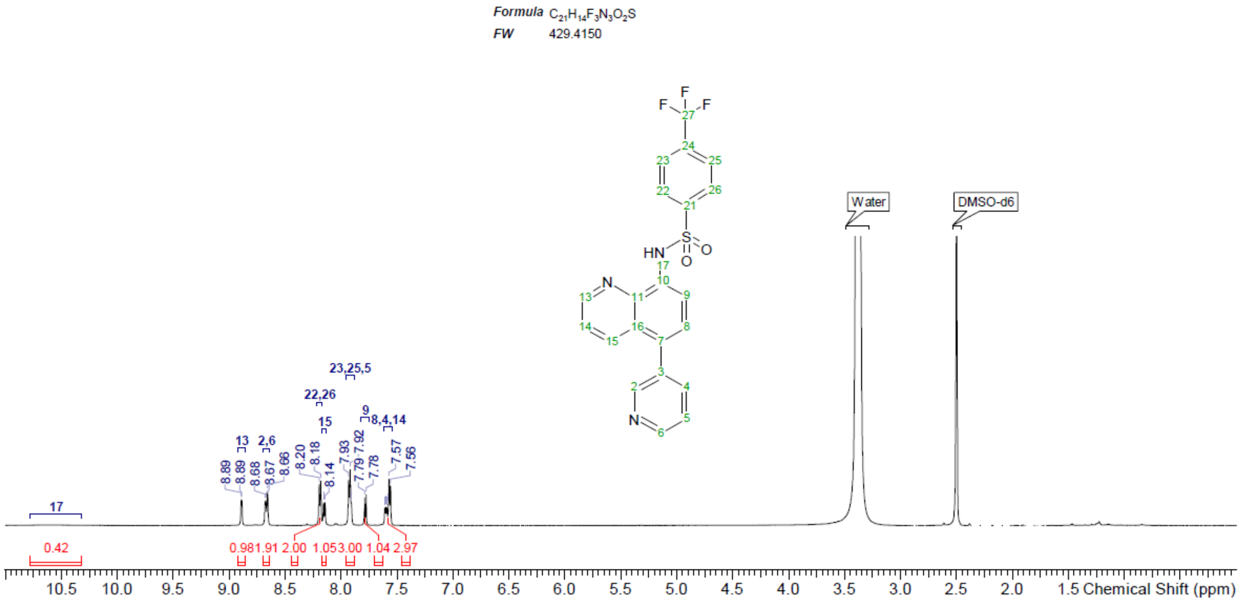

1H NMR spectrum of AK59

Frequency 600.1300 MHz, number of transients 4, original points count 32768, solvent d6-DMSO.

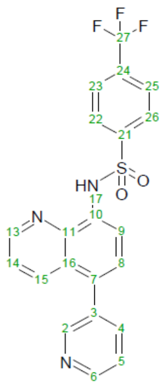

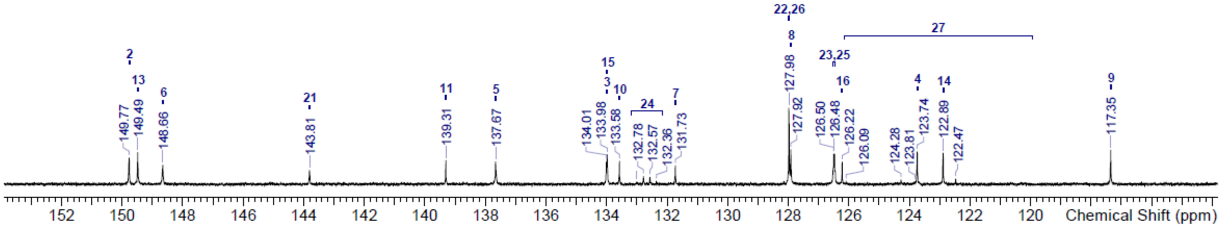

13C NMR spectrum of AK59

Frequency 150.9079 MHz, spectrum offset 17287.3242 Hz, number of transients 512, original points count 65445, solvent d6-DMSO.

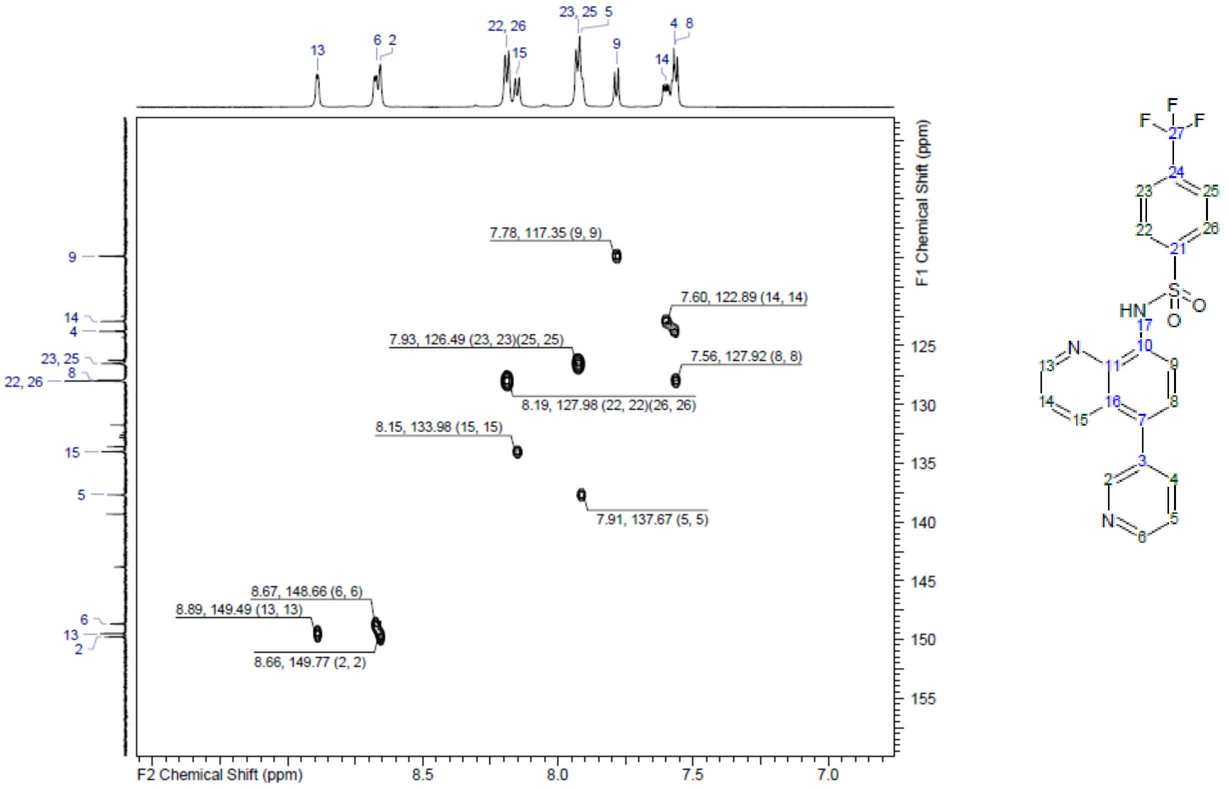

2D HSQC-DEPT NMR spectrum of AK59

Frequency (600.1300, 150.9029) MHz, nucleus (1H, 13C), original points count (1024, 128), solvent d6-DMSO.

F1 Atom (ppm)	F2 Atom	F2 Atom (ppm)	F1 Atom
8.66	2	149.8	2
5.57	4	123.8	4
7.91	5	137.7	5
8.67	6	148.7	6
7.56	8	127.9	8
7.78	9	117.4	9
8.89	13	149.5	13
7.60	14	122.9	14
8.15	15	134.0	15
8.19	22	128.0	22
7.93	23	126.5	23
7.93	25	126.5	25
8.19	26	128.0	26

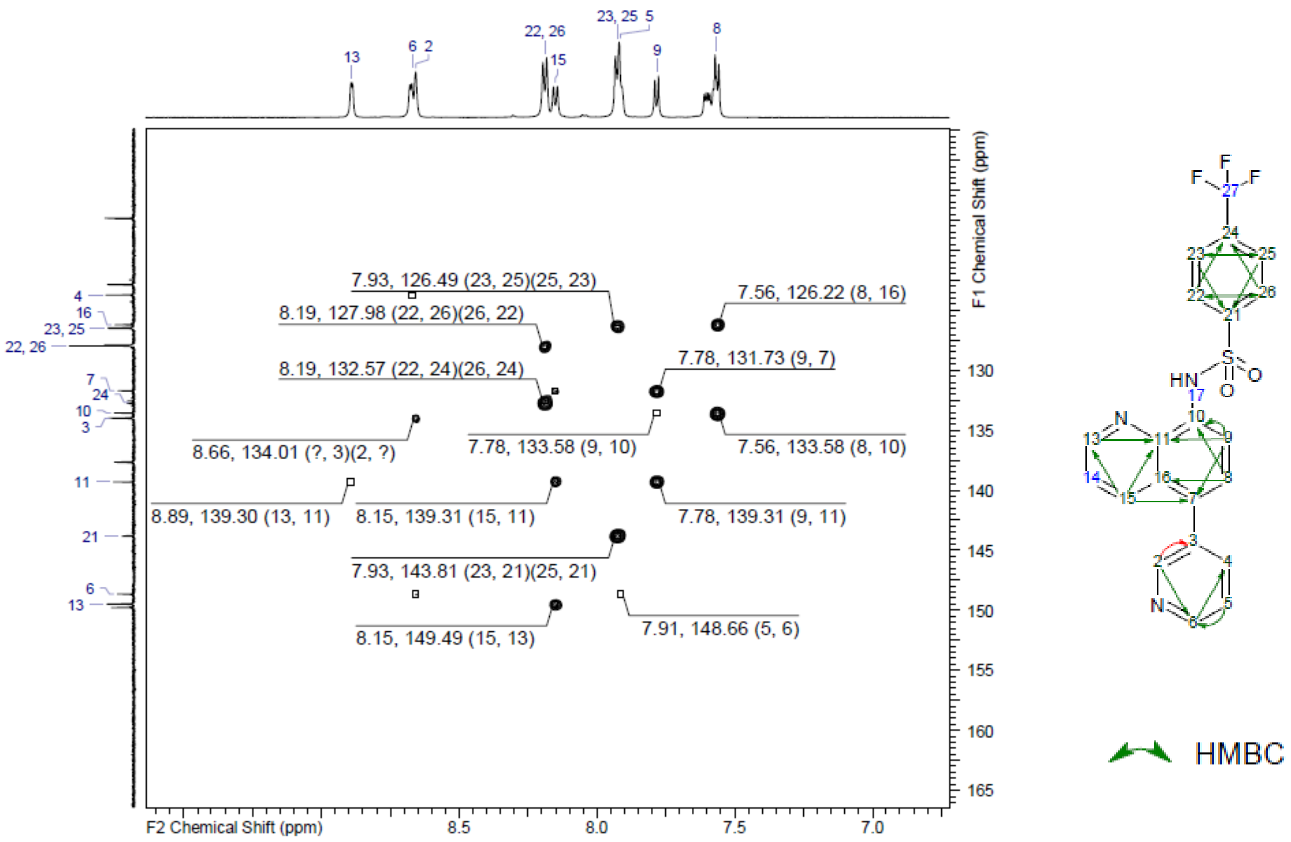

2D HMBC NMR spectrum of AK59

Frequency (600.1300, 150.9029) MHz, nucleus (1H, 13C), original points count (1024, 256), solvent d6-DMSO.

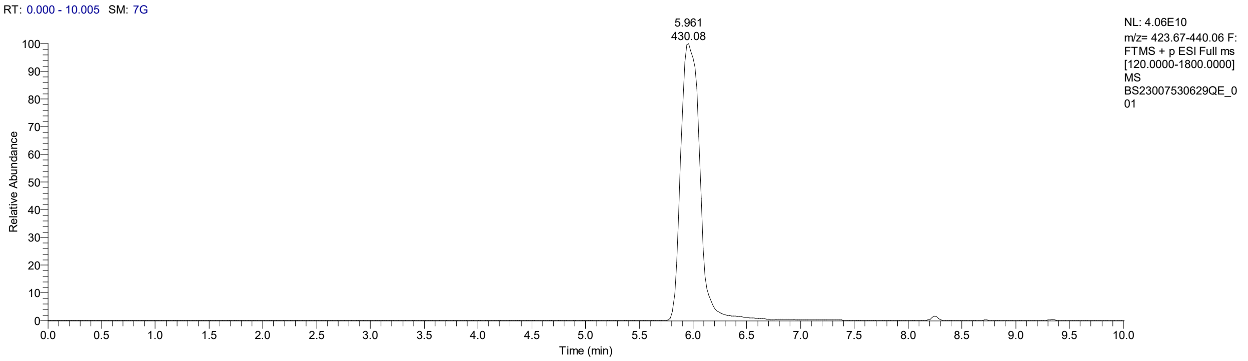

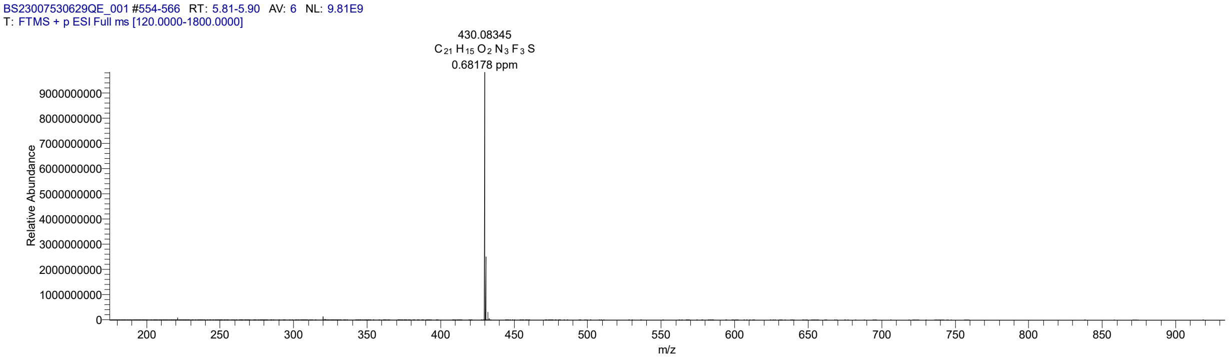

LC-HRMS analysis of AK59

See page 3 and 4 for instrument details.

NMR spectra of QK50

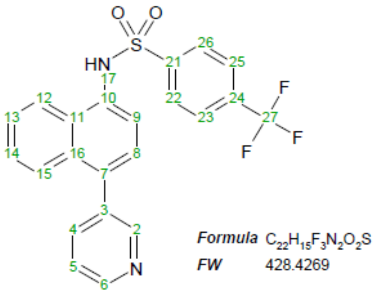

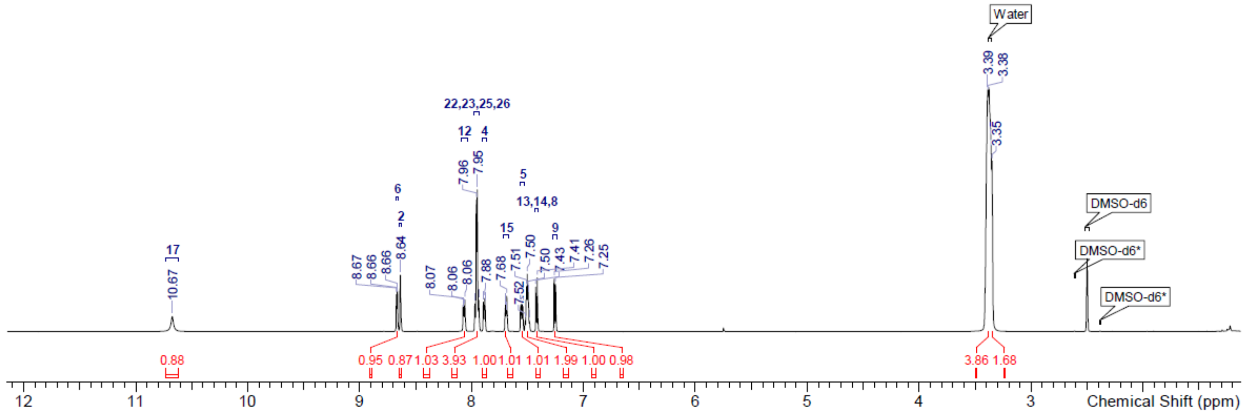

1H NMR spectrum of QK50

Frequency 600.1300 MHz, number of transients 4, original points count 32768, solvent d6-DMSO.

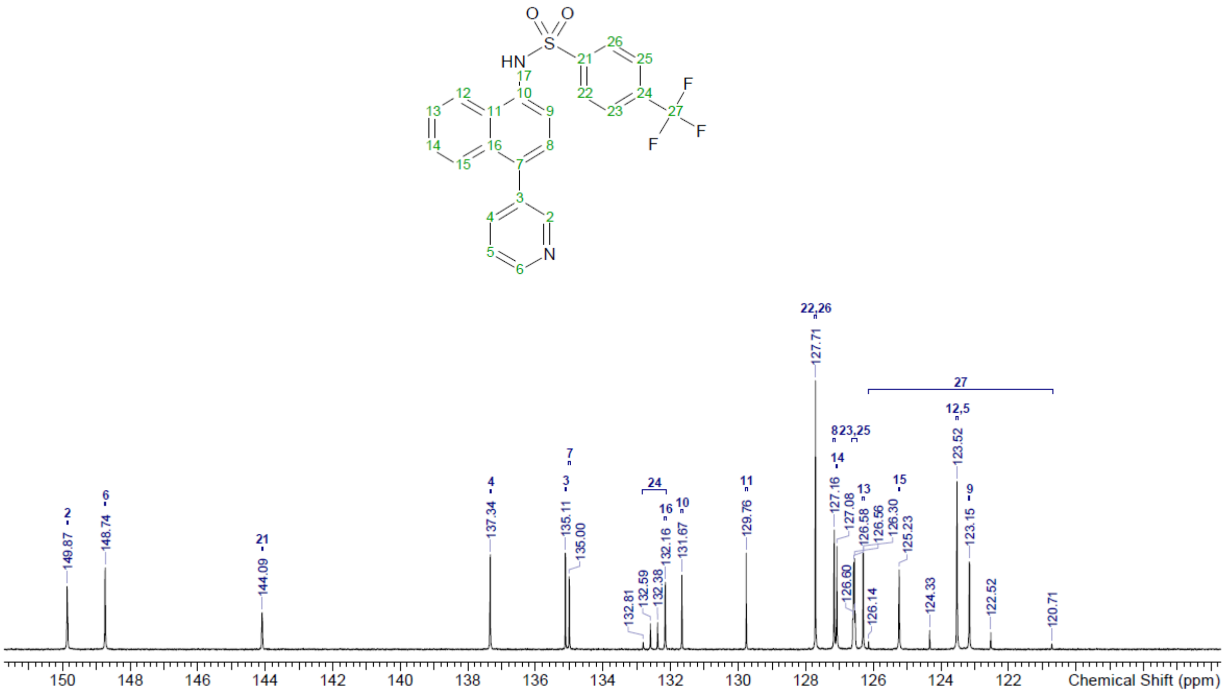

13C NMR spectrum of QK50

Frequency 150.9029 MHz, spectrum offset 17287.5605 Hz, number of transients 2048, original points count 65445, solvent d6-DMSO.

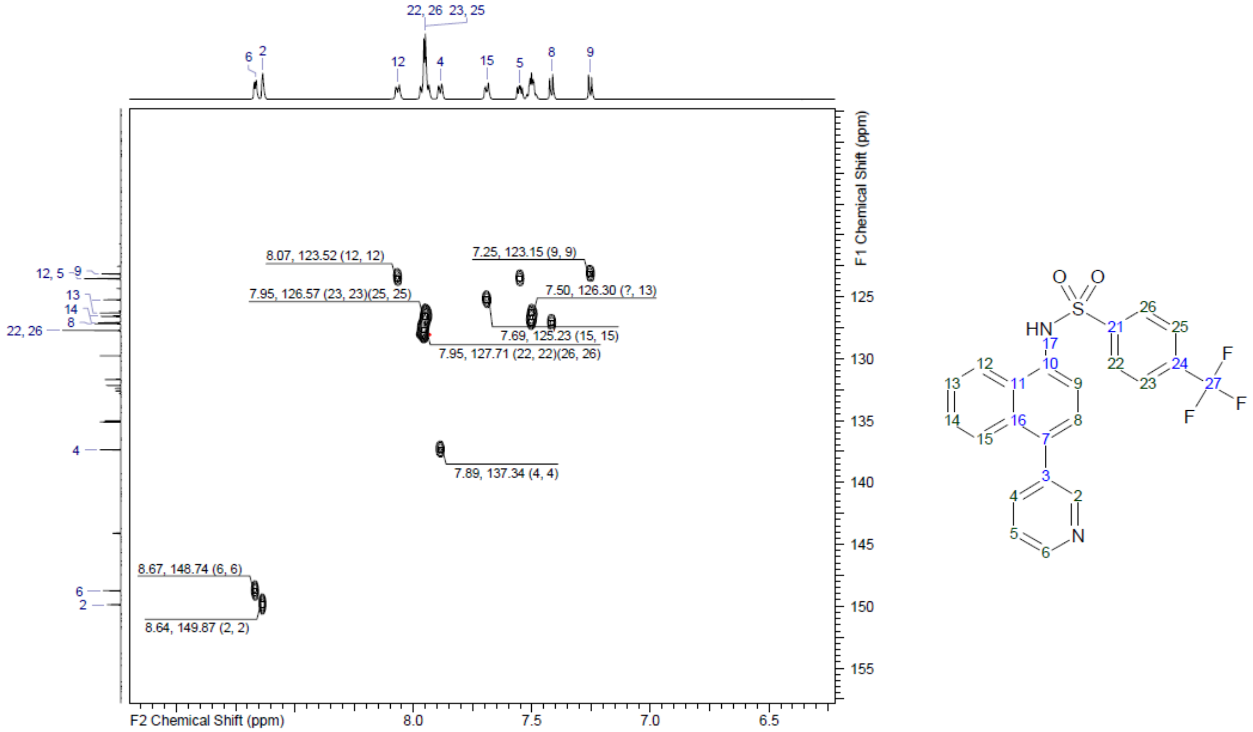

2D HSQC-DEPT NMR spectrum of QK50

Frequency (600.1300, 150.9029) MHz, nucleus (1H, 13C), original points count (1024, 128), solvent d6-DMSO.

F1 Atom (ppm)	F2 Atom	F2 Atom (ppm)	F1 Atom
8.64	2	149.9	2
7.89	4	137.3	4
7.55	5	123.5	5
8.67	6	148.7	6
7.42	8	127.2	8
7.25	9	123.2	9
8.07	12	123.5	12
7.50	?	126.3	13
7.51	?	127.1	14
7.69	15	125.2	15
7.95	22	127.7	22
7.95	23	126.6	23
7.95	25	126.6	25
7.95	26	127.7	26

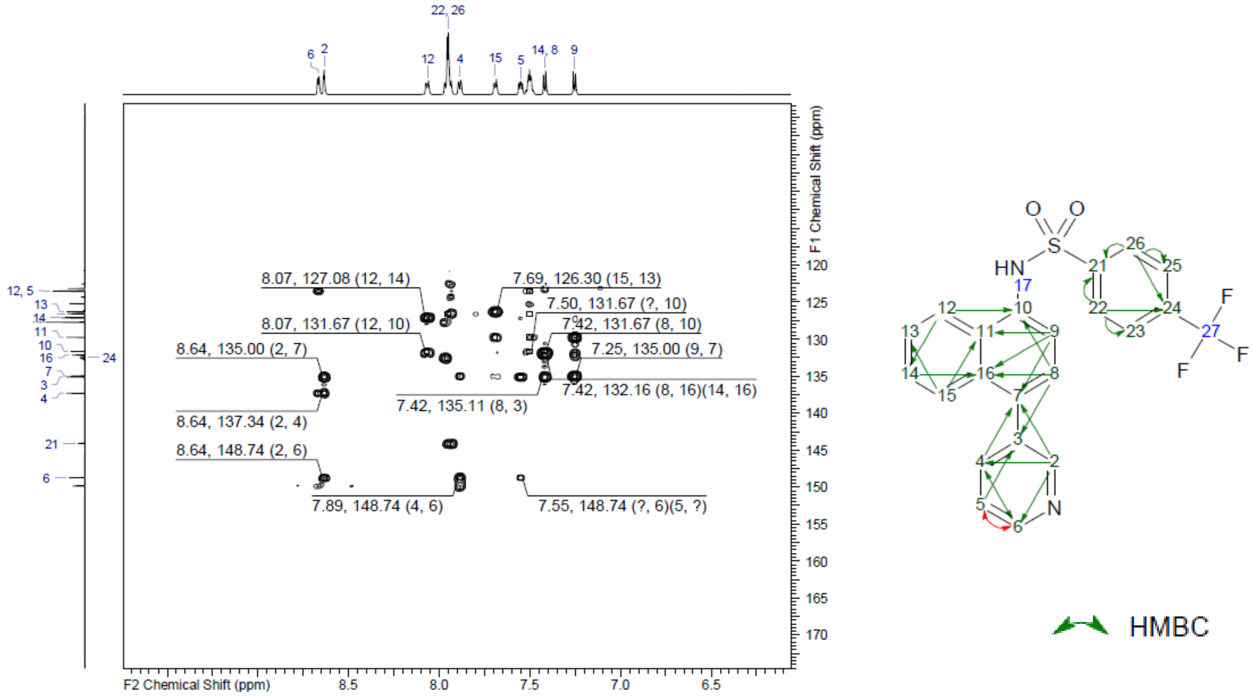

2D HMBC NMR spectrum of QK50

Frequency (600.1300, 150.9029) MHz, nucleus (1H, 13C), original points count (1024, 256), solvent d6-DMSO.

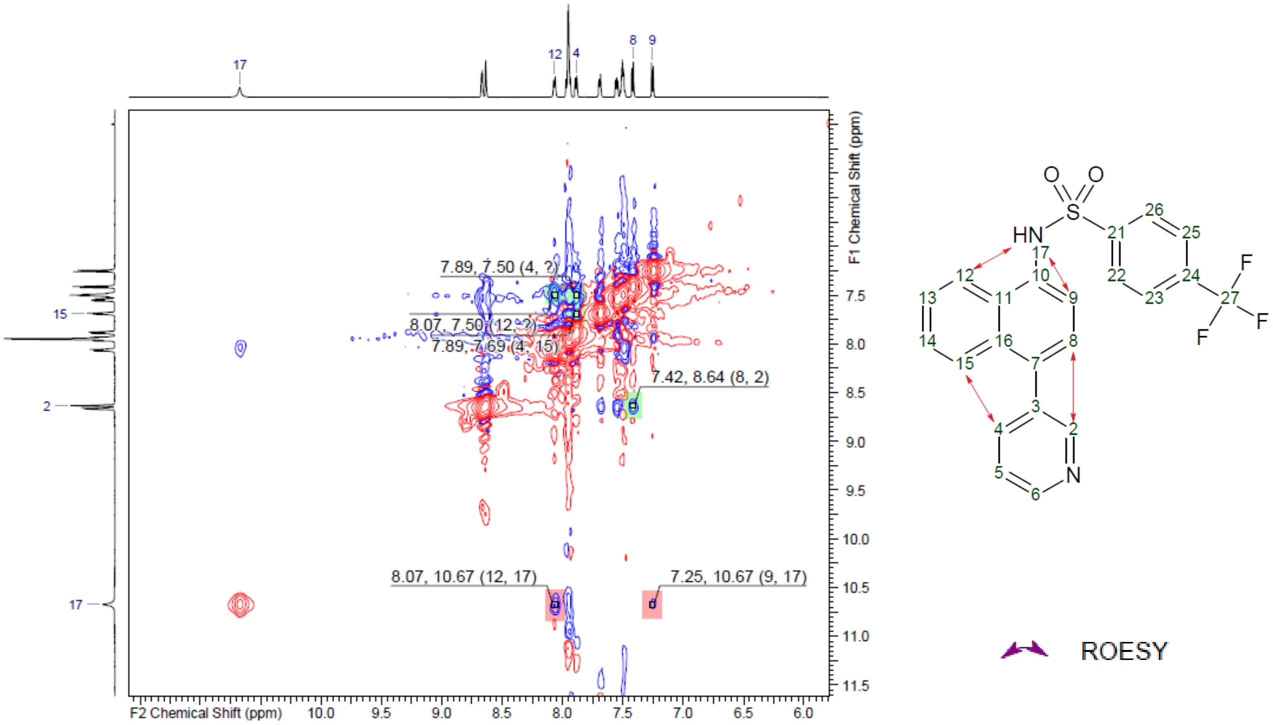

2D ROESY NMR spectrum of QK50

Frequency (600.1300, 600.1300) MHz, nucleus (1H, 1H), original points count (1024, 128), solvent d6-DMSO.

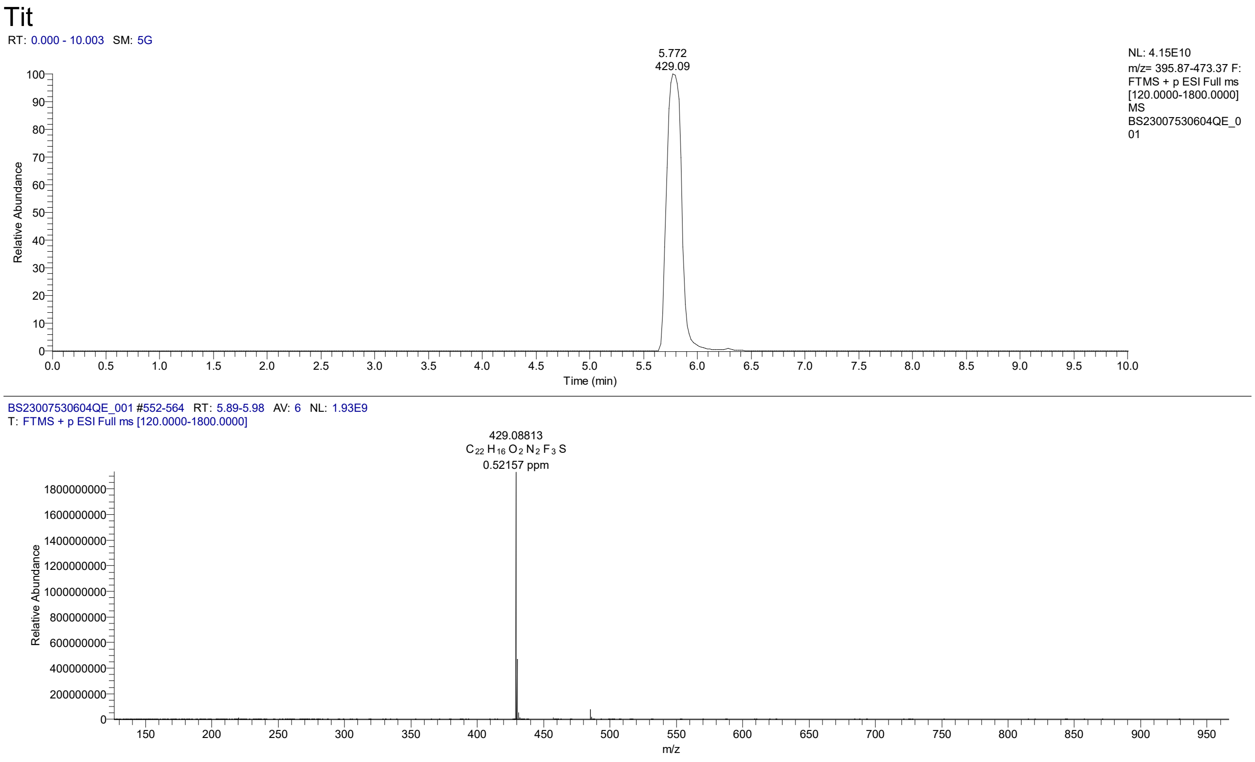

LC-HRMS analysis of QK50

See page 10-12 for instrument details.

Supplementary Table 1. CRISPR sgRNA sequences

Name	Sequence
Ctrl sgRNA	5'-GTAGCGAACGTGTCCGGCGT-3'
HERC4 sgRNA 1	5'-CCAGTCATGAAATAAACCCA-3'
HERC4 sgRNA 2	5'- GTAGACACTGCCCATTATAG-3'
UBA6 sgRNA 1	5'- GCTGAAATGCTGACAAGATG -3'
UBA6 sgRNA 2	5'-GCTGAAATGCTGACAAGATG -3'
UBA5 sgRNA 1	5'- GAGGCGTCCTGTTCTTCCTG-3'
UBA5 sgRNA 2	5'- AGACACAGCAATGCAGAAGA-3'
HERC4 sgRNA 1 TIDE forward primer	5'-GCCATCTGTCTGTTAAATTGTGA-3'
HERC4 sgRNA 1 TIDE reverse primer	5'-TCTCCTGTTACTATGACTCCTCA-3'
HERC4 sgRNA 2 TIDE forward primer	5'-ACTCTAGGCAGCACACTTCT-3'
HERC4 sgRNA 2 TIDE reverse primer	5'-CCTCCCACTGGTATGTAGCC-3'
UBA6 sgRNA 1 TIDE forward primer	5'-GCCCCTTCCTACCTTCCAG-3'
UBA6 sgRNA 1 TIDE reverse primer	5'-GCCAGAACTGAGAAAGCCCT-3'
UBA6 sgRNA 2 TIDE forward primer	5'-TTGTGGCAAATCTGGAGCAC-3'
UBA6 sgRNA 2 TIDE reverse primer	5'-TGCATAATCACAACACCAGAG-3'
UBA5 sgRNA 1&2 TIDE forward primer	5'-GCATTGAAACGAATGGGAAT-3'
UBA5 sgRNA 1&2 TIDE reverse primer	5'-TGGGTGGGGATATGTCTCA-3'

Blots from Supplementary Figures

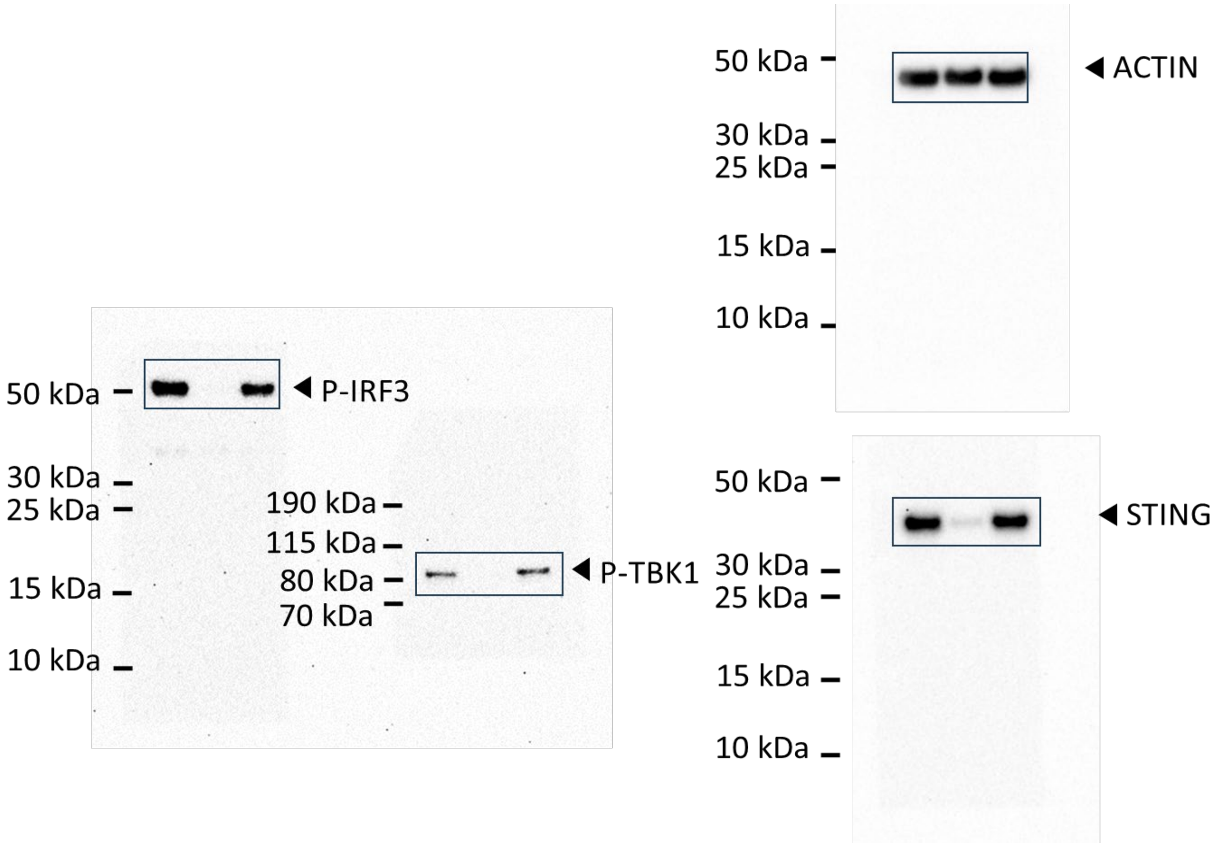

Supplementary Figure 1b

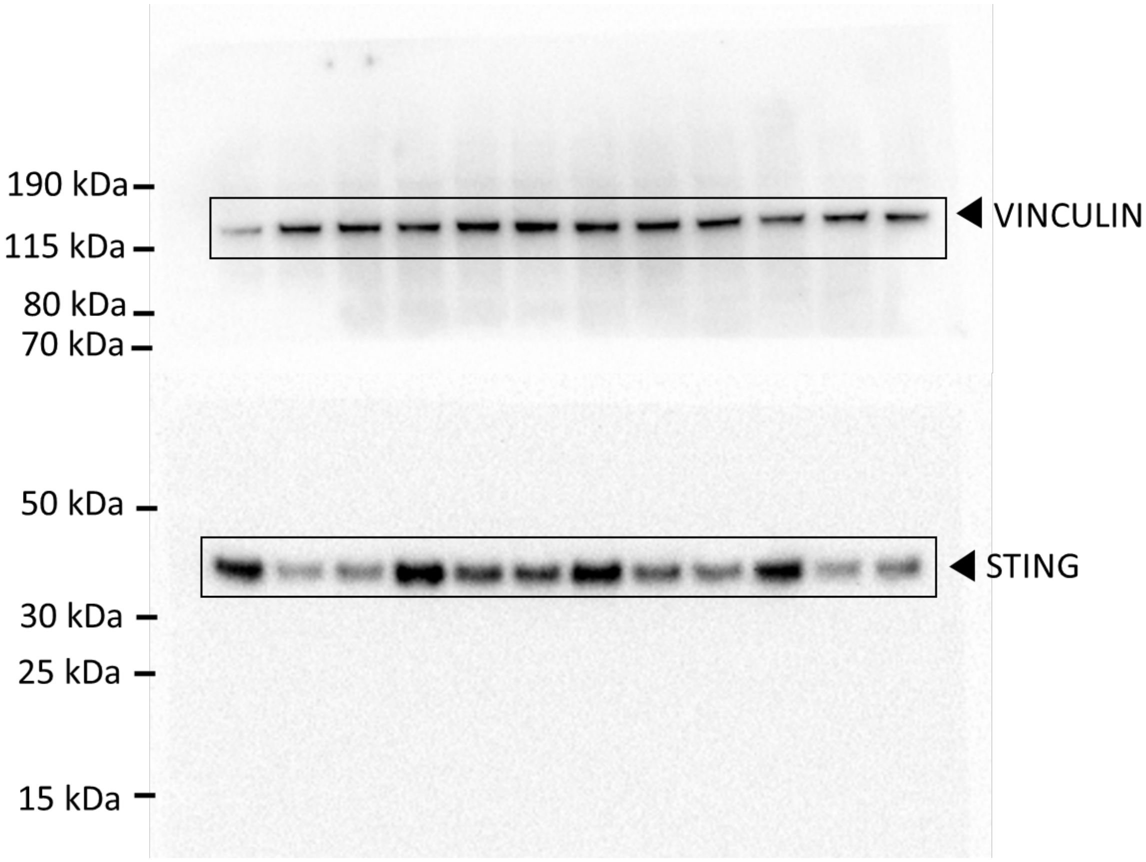

Supplementary Figure 1i

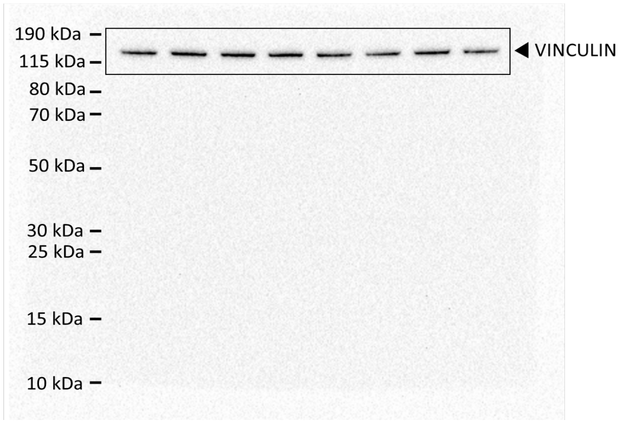

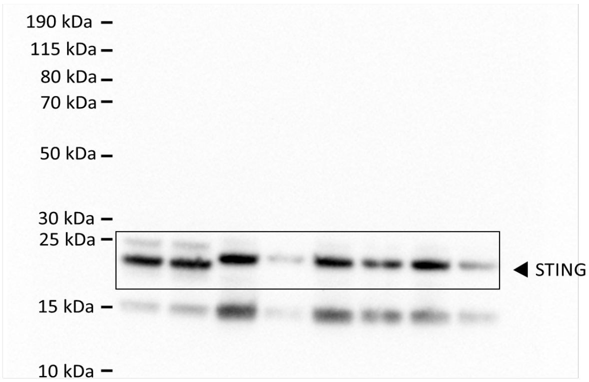

Supplementary Figure 2c

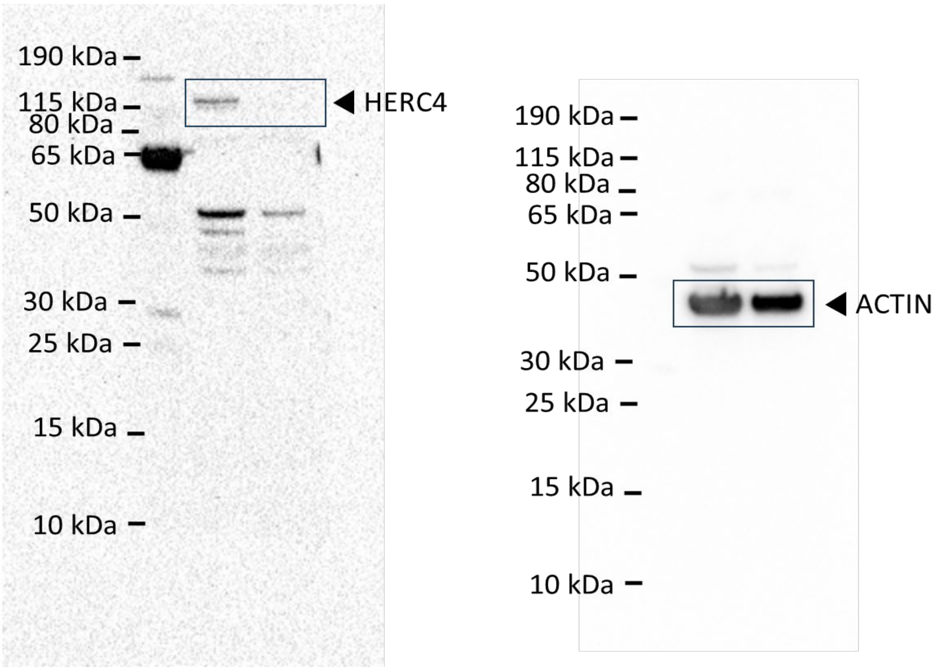

Supplementary Figure 5b

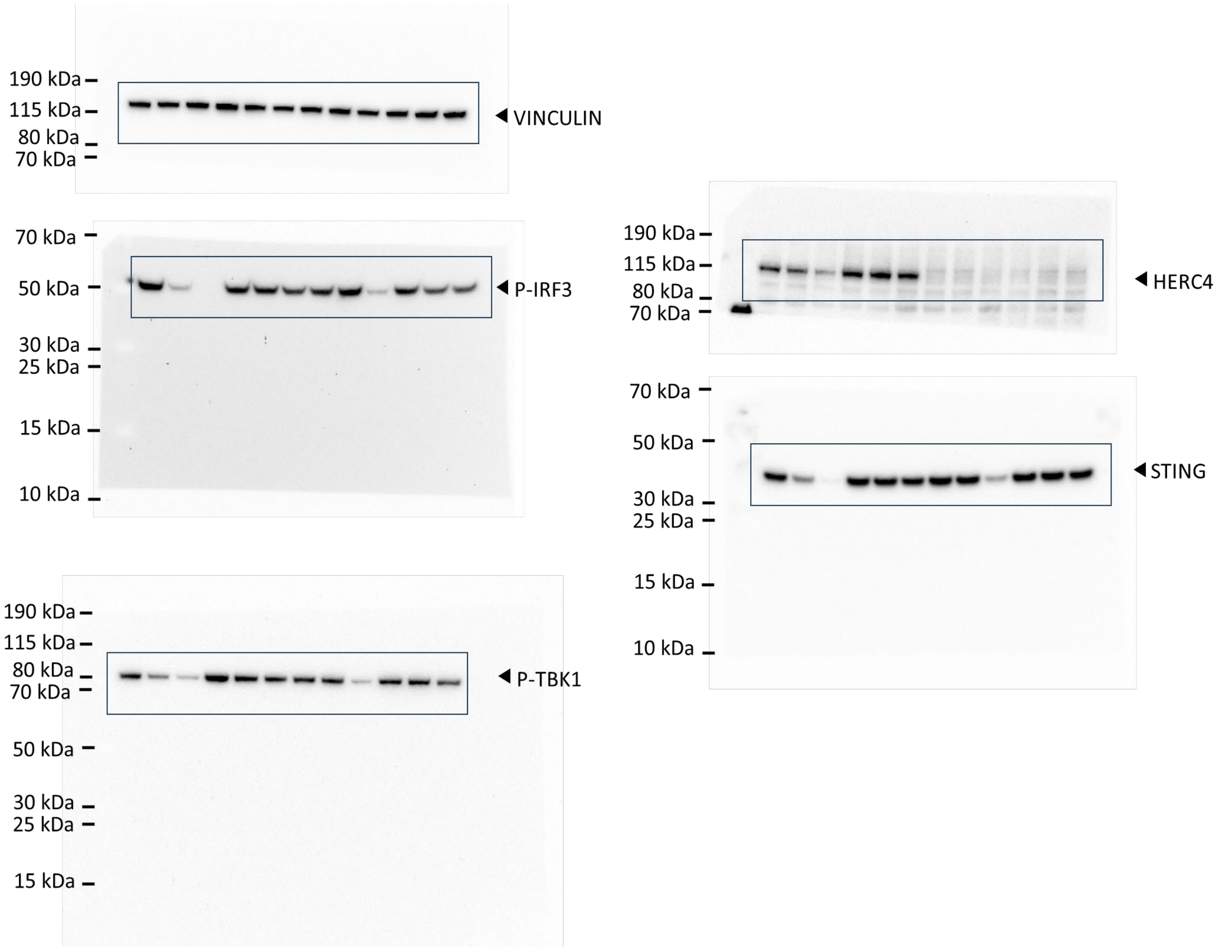

Supplementary Figure 5c

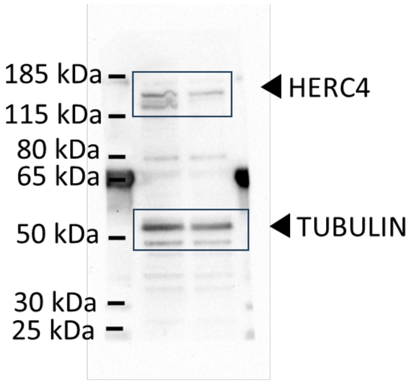

Supplementary Figure 5g

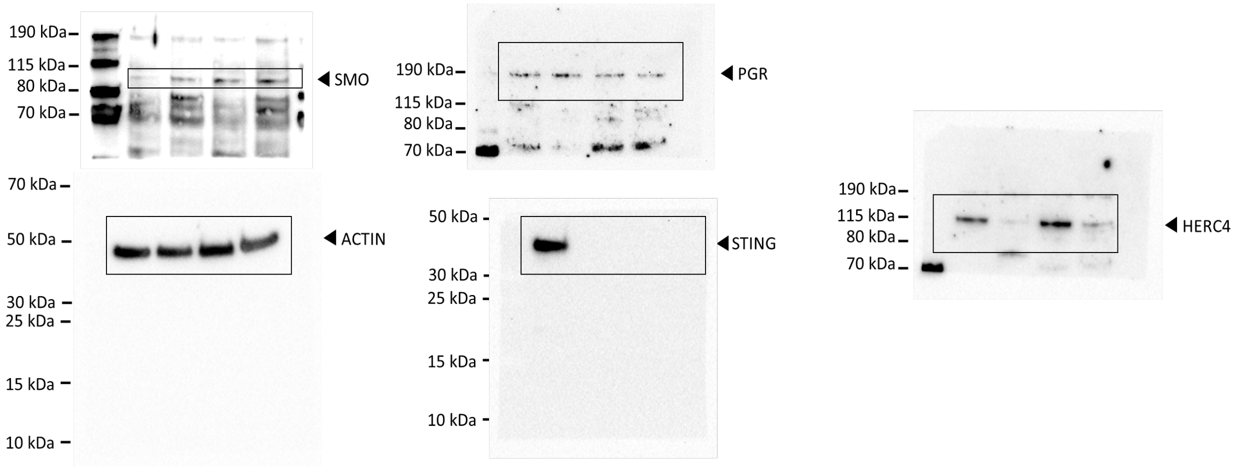

Supplementary Figure 6a

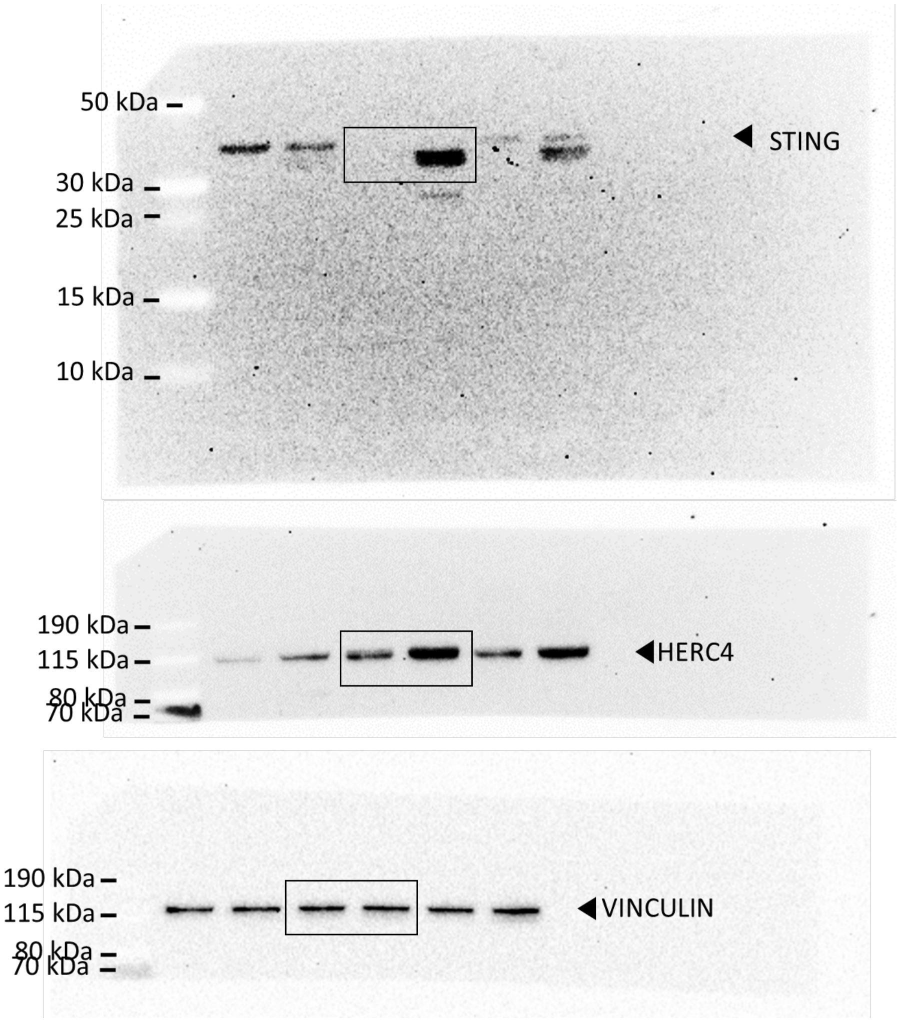

Supplementary Figure 7a

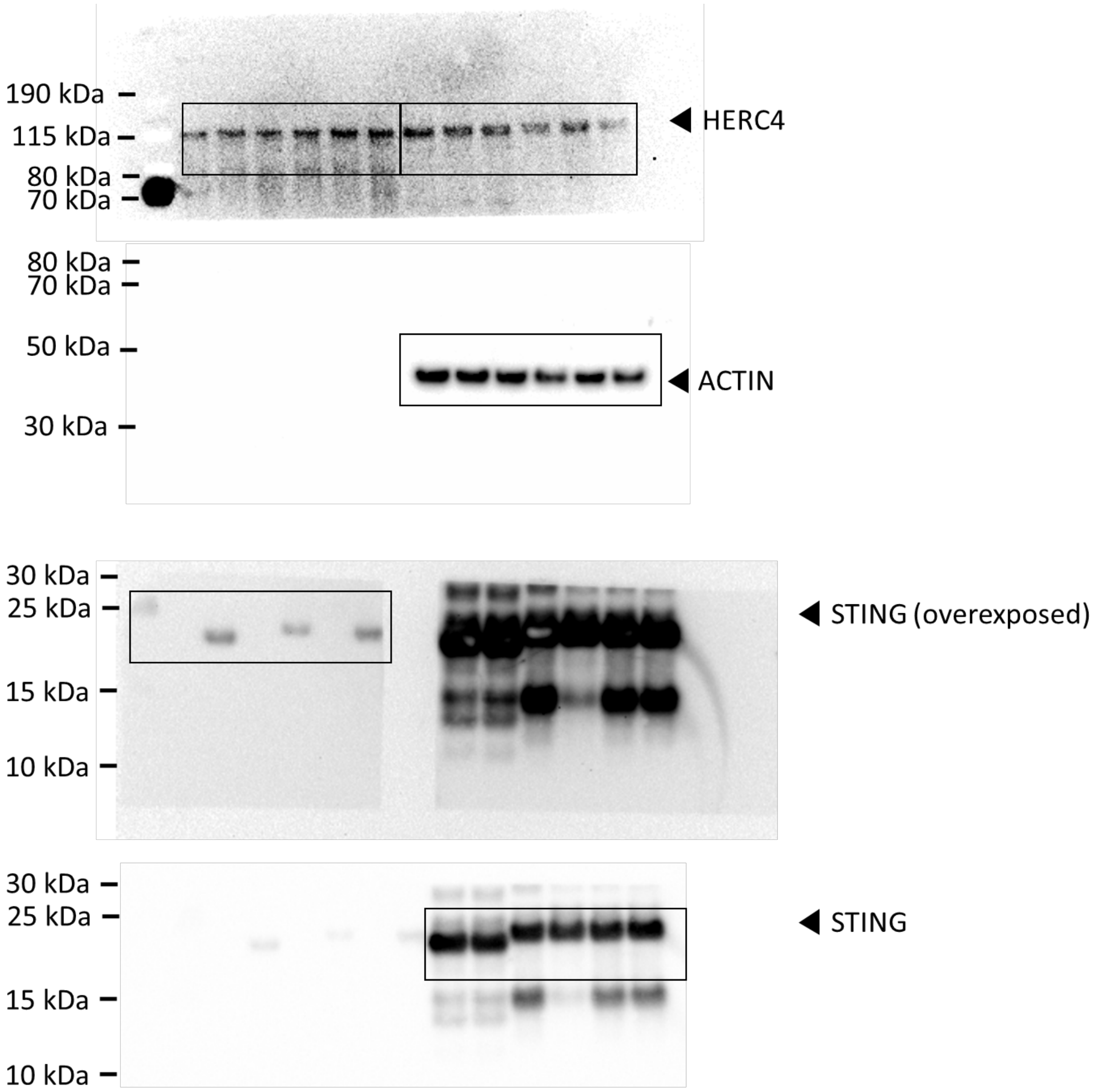

Supplementary Figure 7e
