# Supplementary material for: Small molecule induced STING degradation facilitated by the HECT ligase HERC4
Source: Nat Commun. 2024 May 29;15:4584. doi: 10.1038/s41467-024-48922-w (PMC11137104; doi:10.1038/s41467-024-48922-w)
Supplement: Supplementary file 3 — Description of Additional Supplementary Files [file 41467_2024_48922_MOESM3_ESM.pdf]

File name: Supplementary Data 1
Description: Proteomics results from either 10 μM AK59 or 10 μM QK50 treated THP1 cells.

File name: Supplementary Data 2
Description: CRISPR-Cas9 knockout screen RSA to Q values of hits.

File name: Supplementary Data 3
Description: Proteomics results from 10 μM AK59 treated HERC4 or STING knockout Dual-THP1 cells.

File name: Supplementary Data 4
Description: Vectoral ranking code for prioritizing CRISPR hits. RSA and Q values were utilized in vectoral ranking to access highest difference in comparisons.
