# Supplementary material for: Small molecule induced STING degradation facilitated by the HECT ligase HERC4
Source: Nat Commun. 2024 May 29;15:4584. doi: 10.1038/s41467-024-48922-w (PMC11137104; doi:10.1038/s41467-024-48922-w)
Supplement: Supplementary file 8 — Reporting Summary [file 41467_2024_48922_MOESM8_ESM.pdf]

nature portfolio

Corresponding author(s): Merve Mutlu

Last updated by author(s): Jun 22, 2023

Reporting Summary

Nature Portfolio wishes to improve the reproducibility of the work that we publish. This form provides structure for consistency and transparency in reporting. For further information on Nature Portfolio policies, see our Editorial Policies and the Editorial Policy Checklist.

Statistics

For all statistical analyses, confirm that the following items are present in the figure legend, table legend, main text, or Methods section.

n/a	Confirmed	
☐	☒	The exact sample size (*n*) for each experimental group/condition, given as a discrete number and unit of measurement
☒	☐	A statement on whether measurements were taken from distinct samples or whether the same sample was measured repeatedly
☐	☒	The statistical test(s) used AND whether they are one- or two-sided<br>*Only common tests should be described solely by name; describe more complex techniques in the Methods section.*
☒	☐	A description of all covariates tested
☐	☒	A description of any assumptions or corrections, such as tests of normality and adjustment for multiple comparisons
☐	☒	A full description of the statistical parameters including central tendency (e.g. means) or other basic estimates (e.g. regression coefficient) AND variation (e.g. standard deviation) or associated estimates of uncertainty (e.g. confidence intervals)
☐	☒	For null hypothesis testing, the test statistic (e.g. *F*, *t*, *r*) with confidence intervals, effect sizes, degrees of freedom and *P* value noted<br>*Give P values as exact values whenever suitable.*
☒	☐	For Bayesian analysis, information on the choice of priors and Markov chain Monte Carlo settings
☒	☐	For hierarchical and complex designs, identification of the appropriate level for tests and full reporting of outcomes
☒	☐	Estimates of effect sizes (e.g. Cohen's *d*, Pearson's *r*), indicating how they were calculated

Our web collection on statistics for biologists contains articles on many of the points above.

Software and code

Policy information about availability of computer code

Data collection	EASY-nLC 1200<br>HiSeq 2500 instrument Illumina<br>Fortessa Diva software (version 9.0.1)
Data analysis	Proteome discoverer (version 2.4)<br>R bioconductor (version 4.2 and version 4.0.2)<br>bcl2fastq2 (version 2.17.1.14)<br>fastx-toolkit (version 0.0.13)<br>Flowjo (version 10.6.1)<br>Envision reader workstation (version 1.14.3049.1193)<br>Bio-rad ChemiDoc XRS+ (version 6.0.1)<br>Graphpad prism (version 9)<br>Incucyte (version 2022B rev2)<br>PyMOL Molecular Graphics System (version 3.0 Schrödinger, LLC)

For manuscripts utilizing custom algorithms or software that are central to the research but not yet described in published literature, software must be made available to editors and reviewers. We strongly encourage code deposition in a community repository (e.g. GitHub). See the Nature Portfolio guidelines for submitting code & software for further information.

Data

Policy information about availability of data

All manuscripts must include a data availability statement. This statement should provide the following information, where applicable:

- Accession codes, unique identifiers, or web links for publicly available datasets
- A description of any restrictions on data availability
- For clinical datasets or third party data, please ensure that the statement adheres to our policy

Source data are provided with this paper. The proteomics data generated in this study have been deposited in PRIDE database under accession code PXD040291 (DOI 10.6019/PXD040291) and PXD046677 (DOI 10.6019/PXD046677). Additionally, full list of proteomics results including the raw data counts are provided in Supplementary Table 1 and 3. Protein relative quantification was performed using an in-house developed R (v.4.2) script, available on GitHub (https://github.com/Novartis/px_tmt_daa, doi: 10.5281/zenodo.10962720). Crystal structure of STING was previously published by Lue et al.54 (PDB ID: 7SII). Full list of CRISPR knockout screen results including the raw data is provided in Supplementary Table 2. Vectoral ranking code is provided with this paper.

Research involving human participants, their data, or biological material

Policy information about studies with human participants or human data. See also policy information about sex, gender (identity/presentation), and sexual orientation and race, ethnicity and racism.

Reporting on sex and gender	anonymized
Reporting on race, ethnicity, or other socially relevant groupings	anonymized
Population characteristics	anonymized
Recruitment	Anonymized whole blood was collected under informed consent from healthy volunteers through the Interregionale Blutspende (IRB) of the Swiss Red Cross (SRK) in Bern, Switzerland. Sample collection was approved by Ethikkommission Nordwest- und Zentralschweiz (EKNZ), approval number Req-2017-00050.
Ethics oversight	Samples are anonymized, hence it falls outside of Swiss Human Research Act. No ethics oversight is needed according to Swiss Law for this study.

Note that full information on the approval of the study protocol must also be provided in the manuscript.

Field-specific reporting

Please select the one below that is the best fit for your research. If you are not sure, read the appropriate sections before making your selection.

☒ Life sciences ☐ Behavioural & social sciences ☐ Ecological, evolutionary & environmental sciences

For a reference copy of the document with all sections, see nature.com/documents/nr-reporting-summary-flat.pdf

Life sciences study design

All studies must disclose on these points even when the disclosure is negative.

Sample size	Unless otherwise stated at least three replicates for each experiment were performed. More details can be found in the respective Figure sections, the Method section and the Statistics and Reproducibility section.
Data exclusions	No data exclusions were necessary.
Replication	All experiments were performed at least in three independent replicates.
Randomization	No large dataset where systematic bias could have impacted the data analysis were used in this study. Thus no randomisation was necessary.
Blinding	No analytic bias could be identified in the datasets used in this study and thus blinding was not necessary.

Reporting for specific materials, systems and methods

We require information from authors about some types of materials, experimental systems and methods used in many studies. Here, indicate whether each material, system or method listed is relevant to your study. If you are not sure if a list item applies to your research, read the appropriate section before selecting a response.

Materials & experimental systems

n/a	Involved in the study	
☐	☒	Antibodies
☐	☒	Eukaryotic cell lines
☒	☐	Palaeontology and archaeology
☒	☐	Animals and other organisms
☒	☐	Clinical data
☒	☐	Dual use research of concern
☒	☐	Plants

Methods

n/a	Involved in the study	
☒	☐	ChIP-seq
☐	☒	Flow cytometry
☒	☐	MRI-based neuroimaging

Antibodies

Antibodies used

Rabbit monoclonal anti-STING antibody (1:1000, CST, Cat#13647 or),
Mouse monoclonal anti-STING antibody (1:500, Thermo Fisher Scientific, Cat#MA526030)
Mouse monoclonal anti-UBIQUITIN antibody (1:500, CST, Cat#3936),
Rabbit polyclonal anti-HERC4 antibody (1:500, abcam, Cat#ab856732, batch number GR3184017-12)
Rabbit polyclonal anti-UBA6 antibody (1:1000, CST, Cat#13386)
Rabbit monoclonal anti-UBA5 antibody (1:500, abcam, Cat#ab177478)
Rabbit monoclonal anti-phospho-IRF3 antibody (1:1000, abcam, Cat#76493)
Rabbit monoclonal anti-phospho-TBK1 antibody (1:500, CST, Cat#5483)
Rabbit polyclonal anti-SMO antibody (1:500, abcam, Cat#ab236465)
Rabbit monolconal anti-PGR antibody (1:250, CST, Cat#8757)
Mouse monoclonal anti-ACTIN antibody(1:500, Sigma, Cat#A5441)
Rabbit polyclonal anti-TUBULIN antibody (1:500, CST, Cat#2146)
Rabbit monoclonal anti-VINCULIN antibody (1:500, CST, Cat#13901)
HRP conjugated anti-mouse secondary antibody (1:2500, CST, Cat#7076)
HRP conjugated anti-rabbit secondary antibody(1:2500, CST, Cat#7074)
Amersham ECL prime western blotting detection reagent (Cytiva Life Sciences, Cat#RPN2232)
SuperSignalTM West Femto reagent (Thermo Fisher Scientific, Cat#34094)

Validation

All commercially available, for validation data please see manufacture's website. STING (CST, #13647) validated for western blotting on manufacturer's website (https://www.cellsignal.com/products/primary-antibodies/sting-d2p2f-rabbit-mab/13647). STING (Thermo Fisher, MA526030) validated for western blotting on manufacturer's website (https://www.thermofisher.com/antibody/product/STING-Antibody-clone-OTI4H1-Monoclonal/MA5-26030). UBIQUITIN (CST, Cat#3936) validated for western blotting on manufacturer's website(https://www.cellsignal.com/products/primary-antibodies/ubiquitin-p4d1-mouse-mab/3936?gclid=EAIaIQobChMI56C4wczuhAMVYz4GAB1MxQ6sEAAYAiAAEgLP6fD_BwE&gclsrc=aw.ds). HERC4 (abcam, Cat#ab856732, batch number GR3184017) validated for western blotting on manufacturer's website (https://www.abcam.com/products/primary-antibodies/herc4-antibody-ab85732.html). UBA6 (CST, Cat#13386) validated for western blotting on manufacturer's website (https://www.cellsignal.com/products/primary-antibodies/ube1l2-uba6-antibody/13386). UBA5 (abcam, Cat#ab177478) validated for western blotting on manufacturer's website (https://www.abcam.com/products/primary-antibodies/uba5-antibody-epr11729-ab177478.html). Phospho-IRF3 (abcam, Cat#76493) validated for western blotting on manufacturer's website (https://www.abcam.com/products/primary-antibodies/irf3-phospho-s386-antibody-epr2346-ab76493.html). Phospho-TBK1 (CST, Cat#5483) validated for western blotting on manufacturer's website (https://www.cellsignal.com/products/primary-antibodies/phospho-tbk1-nak-ser172-d52c2-xp-rabbit-mab/5483). SMO (abcam, Cat#ab236465) validated for western blotting on manufacturer's website (https://www.abcam.com/products/primary-antibodies/smoothened-antibody-ab236465.html). PGR (CST, Cat#8757) validated for western blotting on manufacturer's website (https://www.cellsignal.com/products/primary-antibodies/progesterone-receptor-a-b-d8q2j-xp-rabbit-mab/8757). ACTIN (Sigma, Cat#A5441) validated for western blotting on manufacturer's website(). TUBULIN (CST, Cat#2146) validated for western blotting on manufacturer's website (https://www.sigmaaldrich.com/CH/de/product/sigma/a5441). VINCULIN (CST, Cat#13901) validated for western blotting on manufacturer's website (https://www.cellsignal.com/products/primary-antibodies/vinculin-e1e9v-xp-rabbit-mab/13901). HRP conjugated anti-mouse (CST, Cat#7076) validated for western blotting on manufacturer's website (https://www.cellsignal.com/products/secondary-antibodies/anti-mouse-igg-hrp-linked-antibody/7076). HRP conjugated anti-rabbit (1:2500, CST, Cat#7074) validated for western blotting on manufacturer's website (https://www.cellsignal.com/products/secondary-antibodies/anti-rabbit-igg-hrp-linked-antibody/7074).
Additionally, HERC4, UBA5 and UBA6 antibodies were validated using corresponding knockout lines described in the manuscript.

Eukaryotic cell lines

Policy information about cell lines and Sex and Gender in Research

Cell line source(s)

THP1 (ATCC, TIB-202)
THP1-Cas9 Ctrl sgRNA (Source: this study)
THP1-Cas9 HERC4 sgRNA1 (Source: this study)
THP1-Cas9 HERC4 sgRNA2 (Source: this study)
THP1-Cas9 UBA5 sgRNA1 (Source: this study)
THP1-Cas9 UBA5 sgRNA2 (Source: this study)
THP1-Cas9 UBA6 sgRNA1 (Source: this study)
THP1-Cas9 UBA6 sgRNA2 (Source: this study)
Dual-THP1 (Invivogen, thpd-nfis)

	Dual-THP1-Cas9 Ctrl sgRNA (Source: this study)<br>Dua-THP1-Cas9 STING knockout cell line (Source: Willemsen, J. et al. 2021)<br>Dual-THP1-Cas9 HERC4 sgRNA (Source: this study)<br>HEK293T (ATCC, CRL-11268)<br>Jump-In TI 293 (a.k.a. HEK-JumpIN) (Invitrogen, M4455)<br>Jump-In TI 293 Cas9 Ctrl sgRNA (Source: this study)<br>Jump-In TI 293 Cas9 HERC4 sgRNA (Source: this study)<br>primary PBMCs from healthy donors (IRB Switzerland)
Authentication	n/a
Mycoplasma contamination	All the cell lines were tested for mycoplasma contamination and were reported negative.
Commonly misidentified lines<br>(See ICLAC register)	There is no commonly misidentified cell lines were used in this study.

Flow Cytometry

Plots

Confirm that:

☒ The axis labels state the marker and fluorochrome used (e.g. CD4-FITC).

☒ The axis scales are clearly visible. Include numbers along axes only for bottom left plot of group (a 'group' is an analysis of identical markers).

☐ All plots are contour plots with outliers or pseudocolor plots.

☐ A numerical value for number of cells or percentage (with statistics) is provided.

Methodology

Sample preparation	Cells were fixed using 2.5% paraformaldehyde (stock 32%; # 15714-S; Electron Microscopy Sciences) at 37°C for 10 minutes. Cells were then washed using FACS Wash Buffer containing 1x D-PBS + 0.5% FBS + 2 mM EDTA and permeabilized at room temperature for 20 minutes using 100µL of Perm/Wash I (BD # 557885), diluted 1:10 with 1x D-PBS. Cell washing was performed again and then samples were stained with 150µL/sample anti STING Alexa488; 1:200 (anti TMEM173; Abcam # ab198950) diluted in Robosep Buffer, which contained PBS + 2.0% FBS + 1 mM EDTA (Stemcell; # 20104) at 4oC for 1-2 hours. After antibody incubation, cells were washed with Wash Buffer three times then cell pellets were resuspended in FACS Wash Buffer and flow cytometry acquisition on Fortessa was then performed. Analysis was performed using FlowJo software (version 10.6.1)
Instrument	BD LSRFortessa X20 Cell analyzer
Software	Diva software, Flowjo software (version 10.6.1)
Cell population abundance	For more accurate quantification we aimed to get single cells to measure the STING-FITC signal per cell. Total population of single cells after gating were taken into account.
Gating strategy	Single cells were gated with first FSC-A vs. SSC-A scatter plot. Gated cells were then further gated with FSC-A vs. FSC-H and SSC-A vs. SSC-H to make sure each event is a single cell.

☒ Tick this box to confirm that a figure exemplifying the gating strategy is provided in the Supplementary Information.
